# Unveiling Organic Electrode Materials in Aqueous Zinc-Ion Batteries: From Structural Design to Electrochemical Performance

**DOI:** 10.1007/s40820-024-01404-6

**Published:** 2024-05-14

**Authors:** Dujuan Li, Yuxuan Guo, Chenxing Zhang, Xianhe Chen, Weisheng Zhang, Shilin Mei, Chang-Jiang Yao

**Affiliations:** State Key Laboratory of Explosion Science and Safety Protection, School of Mechatronical Engineering, Beijing Institute of Technology, Beijing, 100081 People’s Republic of China

**Keywords:** Aqueous zinc-ion batteries, Organic electrodes, Functional groups, Molecular size/geometry, Electrochemical performances

## Abstract

Aqueous zinc-ion batteries (AZIBs) are one of the most compelling alternatives of lithium-ion batteries due to their inherent safety and economics viability. In response to the growing demand for green and sustainable energy storage solutions, organic electrodes with the scalability from inexpensive starting materials and potential for biodegradation after use have become a prominent choice for AZIBs. Despite gratifying progresses of organic molecules with electrochemical performance in AZIBs, the research is still in infancy and hampered by certain issues due to the underlying complex electrochemistry. Strategies for designing organic electrode materials for AZIBs with high specific capacity and long cycling life are discussed in detail in this review. Specifically, we put emphasis on the unique electrochemistry of different redox-active structures to provide in-depth understanding of their working mechanisms. In addition, we highlight the importance of molecular size/dimension regarding their profound impact on electrochemical performances. Finally, challenges and perspectives are discussed from the developing point of view for future AZIBs. We hope to provide a valuable evaluation on organic electrode materials for AZIBs in our context and give inspiration for the rational design of high-performance AZIBs.

## Highlights

- A comprehensive introduction into organic cathode materials for aqueous zinc-ion batteries with specific focus on their structural–property relationship based on the variations in composition, geometry, and molecular size.
- For each representative organic cathode, the unique electrochemistry has been discussed to provide insight into the underlying working mechanism.
- Summarized pros and cons of different organic cathodes and outlined challenges plus future research directions.

## Graphical abstract

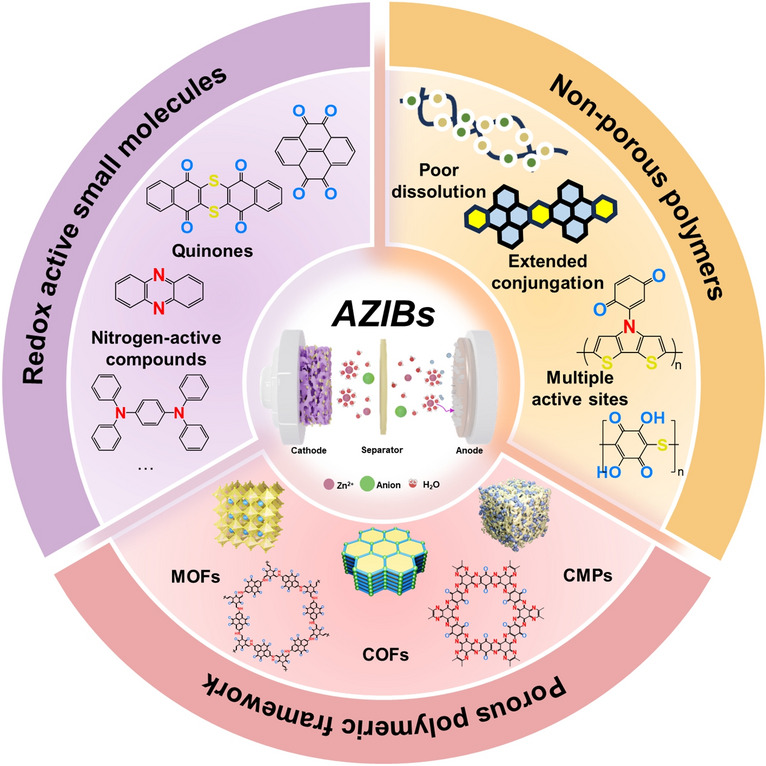

## Introduction

**Fig. 1:**
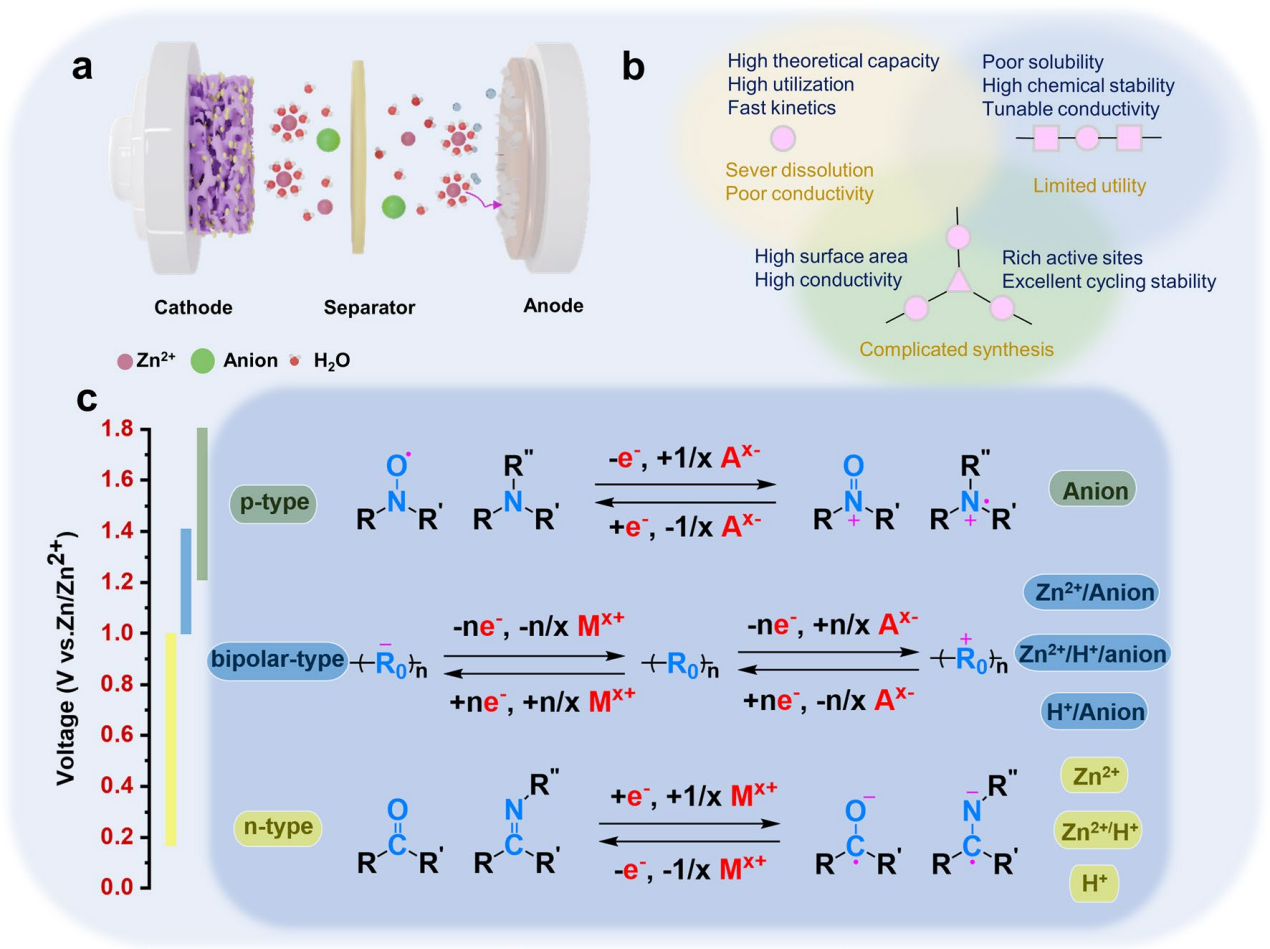
**a** Diagram of aqueous zinc-ion battery structure and ion storage. **b** Characteristics of three different dimensions of organic materials. **c** Energy storage mechanism of p-type, bipolar and n-type electrodes

In light of the increasing resource scarcity, escalating environmental pollution concerns, and a rising number of battery safety issues worldwide, there is a growing demand for more stringent criteria in assessing secondary batteries. The eco-friendliness of secondary batteries has now become a pivotal consideration [1, 2]. In response to this, a strategic change has emerged, where traditional metal-ion battery negative electrodes are being replaced with environmentally safer metals, and organic electrolytes are being substituted with aqueous electrolytes. Aqueous rechargeable metal-ion batteries are a perfect fit for this innovative strategy. The low cost, high safety, and environmental friendliness of this electrochemical energy storage battery make it a promising option for sustainable development [3–5]. Importantly, the ionic conductivity of aqueous electrolytes is two orders of magnitude higher than that of non-aqueous electrolytes [6–8]. So far, various lithium-rich alkali metal cations [9–12] (such as Na^+^ and K^+^) [13] and multivalent charges [14–18] (such as Mg^2+^, Al^3+^, and Zn^2+^) have been developed. Redox reactions in multivalent ionic systems, which involve multiple electrons, can potentially achieve higher energy densities [19–21]. However, not all high-valent metal ions are suitable for the charge and discharge processes. For instance, the slow diffusion of Mg^2+^ within the host lattice and the passivation of the Mg anode significantly impede the transport of Mg^2+^ ions [22–25]. In aqueous aluminum-ion batteries (AIBs), electrolyzing the anode in an aqueous electrolyte (4 < pH < 8) leads to the formation of Al_2_O_3_, accompanied by a reduction in electrode potential. The uneven corrosion of aluminum in the electrochemical reaction has limited the availability of relevant reports [26–29]. Compared to aluminum and magnesium anodes, zinc anodes exhibit a greater stability and a favorable redox potential of 0.76 V vs. SHE. Zinc is abundant in nature, possessing a high volumetric energy density (5855 mAh cm^−3^), and is well-suited for utilization with water. The slightly acidic or neutral electrolyte further enhances their potential for extensive energy storage and various other applications [30–34]. These advantages have led scientists to investigate the internal solvation and ion storage of AZIBs (Fig. 1a).

However, the state-of-the-art AZIBs are far from commercialization due to the remaining challenges including but not limited to following issues: (1) traditional aqueous electrolytes struggle with high-voltage operation and low-temperature conditions; (2) zinc anodes are prone to corrosion passivation and dendrite formation; (3) cathode degradation and parasitic reactions lead to low capacity and short battery life. To address these issues, three main approaches are being pursued to enhance AZIBs performance: exploring and modifying zinc anodes [35–43], optimizing electrolytes [44–49], and constructing highly conductive electrodes [50]. Traditional rigid inorganic electrode materials used in AZIBs have limited molecular design space and can suffer irreversible damage when ions are inserted and extracted within their crystal lattices. In comparison, organic materials provide a solution to this issue [32, 51]. The rich designability and flexibility of organic electrode materials enable them to have abundant redox sites and excellent reversibility. Many reviews on organic electrodes in AZIBs have been published in the past years, either to describe the mechanism according to the classification of redox-active sites, proposing specific strategies based on the underlying electrochemistry to improve the battery performances. These reviews certainly provide a systematic survey, however neglecting the physical/physicochemical effects derived from the molecular geometry.

Apart from active functional groups, manipulation on the macroscopic molecular size/geometry of organic materials is of great significance. As shown in Fig. 1b, the exposure of active sites in organic small molecules has promoted the higher utilization of active functional groups, leading to fast kinetics and higher initial specific capacity [52, 53]. However, the intrinsic dissolving problem of organic small molecules and their discharge products in water has hindered their practical applications. The chemical polymerization of organic small molecules into linear polymers reduces their water solubility significantly, resulting in a substantial improvement in cycle stability [54, 55]. Besides, due to the connectedness of monomers in polymers, electron transport may be more effective than in small molecules. Nevertheless, the irregular arrangement of polymers often encases the active sites of the molecules, leading to a noticeable decrease in specific capacity and electrical conductivity. As a solution, researchers have turned their attention to the effect of monomer joint, incorporating groups with enhanced conjugation during polymerization to boost molecular planarity and electrical conductivity. This approach has given rise to conjugated organic frameworks (COFs), conjugated porous polymers (CMPs) and metal–organic frameworks (MOFs). These materials exhibit regular planar conjugated structures and porous structures, which further aid in boosted electron and ion transport, respectively [56, 57]. Nonetheless, the synthesis conditions can be harsh, and the presence of a large conjugated structure often results in so-called dead mass.

Therefore, we discuss the design principle of organic molecules with emphasis not only on active centers but also molecular size/geometry, highlighting the importance of integrating strategies. Progresses of organic electrode materials for AZIBs up to date have been summarized, and possibilities for rational design of organic electrode materials for AZIBs have been discussed in perspectives.

## Energy Storage Mechanism

Organic electrode materials in AZIBs can be classified into n-type, p-type, or bipolar materials according to the redox processes and the type of binding ions (Fig. 1c) [58, 59]. For n-type organics, redox reactions occur between neutral and negatively charged states, initially undergoing a reduction reaction combined with cations [59]. These electrodes generally include quinones, imides, and phenazines. P-type materials typically undergo an oxidation reaction first and bind to anions in the electrolyte to remain electrically neutral [60], typically covering trianiline derivatives, nitronyl nitroxide, and other compounds. In addition to the above single-polar electrodes, bipolar process means that the electrodes are reduced to bind cations during discharge and oxidized to bind anions during charging [61].

It is noteworthy that the p-type materials generally possess higher redox potential than n-type materials, which is beneficial to approaching high energy density. The redox kinetics of p-type materials are typically faster than that of n-type materials because it does not involve the rearrangement of chemical bonds. Typically, n-type organic materials can be used as either anodes or cathodes, according to their actual redox potential. Compared with the large dead mass of p-type materials, abundant redox sites of n-type materials determine their high capacities. Bipolar materials that absorb the high-capacity characteristics of n-type and the high-voltage characteristics of p-type may have more advantages in comprehensive performance. However, the restricted voltage range of aqueous electrolytes limits the utilization of certain high-voltage p-type sites in traditional low-voltage systems, which means the design of molecular structures and active sites should align with the requirement of the battery system [62].

Apart from the redox reaction mechanism of AZIBs, charge carriers are also the focus of attention. Protons are present in most commonly used electrolytes of AZIBs such as ZnSO_4_, Zn(CF_3_SO_3_)_2_, and Zn(ClO_4_)_2_. In dozens of reports on n-type cathodes, H^+^/Zn^2+^ co-insertion/extraction mechanism, Zn^2+^ insertion/extraction mechanism, and H^+^ insertion/extraction mechanism have been proposed. Although there is no direct theory to explain what kind of molecules can storage H^+^, it is clear from the current research that protons and zinc ions are competitive, undoubtedly accompanying with advantages and disadvantages. On the one hand, the size of H^+^ is the smallest, which is beneficial to fast storage and rate performance; on the other hand, the embedding and removal process of H^+^ affects the acid alkalinity near the zinc anode and accelerates corrosion, resulting in poor stability. The typical problem with Zn^2+^ storage in n-type is its size and strong electrostatic action, which results in slow kinetics. In many two-dimensional inorganic electrodes, it is necessary to expand the material spacing to minimize the energy barriers. When storing larger anions, the reversibility of the structure needs to be considered.

## Organic Small Molecules for Efficient Energy Storage

Redox-active small molecules, which were initially used in aqueous zinc-ion batteries, are appreciated for their ease of preparation, rapid kinetics, high capacity, and easily adjustable structures. In this section, we systematically delve into the study of organic small molecule electrodes in AZIBs, categorizing them into three types based on their active sites: quinones, imides, and nitrogen-containing species. Furthermore, we provide a summary of the electrochemical properties of typical small redox molecules (Table 1 and Fig. 2) to facilitate straightforward comparisons.

**Table 1:** Summary of electrochemical performance of redox-active small molecules

#	Charge carriers	Cathode	Anode	Electrolyte	Output voltage(V)	Cycling stability	Specific capacity (mAh g^−1^)	Refs
1	Al^3+^/Mg^2+^/Zn^2+^	Al/Mg/Zn	9,10-AQ	1 M Al_2_(SO_4_)_3_/2 M MgSO_4_/2 M ZnSO_4_	/	Al^3+^: 94.5%/500/800 mAh g^−1^	Al^3+^: 211.4 at 800 mA g^−1^	[63]
2	Zn^2+^	C4Q	Zn	3 M Zn(CF_3_SO_3_)_2_	~ 1.0	87%/1000/500 mA g^−1^	335 at 20 mA g^−1^	[64]
3	Zn^2+^	PTO	Zn	2 M ZnSO_4_	0.8 ~ 1.0	70%/1000/3 A g^−1^	336 at 0.04 A g^−1^	[65]
4	Zn^2+^	TABQ	Zn	1 M ZnSO_4_	~ 0.9	~ 100%/1000/5 A g^−1^	303 at 0.1 A g^−1^	[66]
5	Zn^2+^	PQ-Δ	Zn	3 M Zn(CF_3_SO_3_)_2_	~ 0.84	Keep constant/500/150 mA g^−1^	225 at 30 mA g^−1^	[67]
6	Zn^2+^ and H^+^	DTT	Zn	2 M ZnSO_4_	~ 0.8	83.8%/23000/50 mA g^−1^	210.9 at 50 mA g^−1^	[68]
7	Zn^2+^	APh-NQ@CNT	Zn	2 M Zn(CF_3_SO_3_)_2_	~ 0.65	67.8%/1000/100 mA g^−1^	202 at 100 mA g^−1^	[69]
8	H^+^	4S6Q	Zn	3.5 M Zn(ClO_4_)_2_	~ 0.8	Keep constant/20000/3 A g^−1^	240 at 150 mA g^−1^	[70]
9	Zn^2+^	1,4,5,8-naphthalene diimide (NDI)	Zn	2 M ZnSO_4_ and 0.5 M Na_2_SO_4_	~ 0.6	76%/5000/3 A g^−1^	200 at 50 mA g^−1^	[71]
10	Zn^2+^	π-PMC	Zn	2 M ZnCl_2_	~ 0.5	80.8%/500/8 A g^−1^	122.9 at 0.2 A g^−1^	[72]
11	Zn^2+^	RF	Zn	3 M Zn(CF_3_SO_3_)_2_	~ 0.6	92.7%/3000/5 A g^−1^	145.5 at 0.01 Ag^−1^	[73]
12	Zn^2+^	DC-PDESA	Zn	3 M Zn(CF_3_SO_3_)_2_ − 40% HBCD − 60% H_2_O electrolyte	1.4	98.3%/2000/1.48 A g^−1^	121 at 0.2 C (1.0 C = 148 mA g^−1^)	[74]
13	Zn^2+^	PNZ	Zn	2 M ZnSO_4_	~ 0.9	79%/1000/1 A g^−1^	232 at 20 mA g^−1^	[75]
14	Zn^2+^	DQP	Zn	1 M ZnSO_4_	0.81	86%/1000/5 A g^−1^	413 at 50 mA g^−1^	[76]
15	H^+^	HATN	Zn	2 M ZnSO_4_	~ 0.6	93.3%/5000/5 A g^−1^	405 at 100 mA g^−1^	[77]
16	Zn^2+^ and H^+^	HATN-3CN	Zn	2 M ZnSO_4_	~ 0.71	90.7%/5800/5 A g^−1^	320 at 0.05 A g^−1^	[78]
17	Zn^2+^ and H^+^	HATN-PNZ	Zn	2 M ZnSO_4_	0.6–0.8	0.14% decay rate/45000/50 A g^−1^	257 at 5 A g^−1^	[79]
18	Zn^2+^ and H^+^	TAP/Ti_3_C_2_T_x_	Zn	2 M ZnSO_4_	0.6–0.8	81.6%/10000/0.04 A g^−1^	303 at 0.04 A g^−1^	[80]
19	Zn^2+^ and H^+^	HATNQ	Zn	3 M ZnSO_4_	~ 0.7	0.0068% per cycle/11000/5 A g^−1^	482.5 at 0.2 A g^−1^	[81]
20	Zn^2+^ and H^+^	HOF-HATN	Zn	1 M ZnSO_4_	~ 0.6	88%/10000/5 A g^–1^	320 at 50 mA g^–1^	[82]
21	Zn^2+^ and H^+^	TPHATP	Zn	2 M ZnSO_4_	~ 0.7	97.44%/5000/10 A g^–1^	318.3 at 0.1 A g^–1^	[83]
22	Zn^2+^ and H^+^	TAPQ	Zn	1 M ZnSO_4_	~ 0.9	92%/250/50 mA g^–1^	472 at 50 mA g^–1^	[84]
23	Zn^2+^ and H^+^	TQD	Zn	4 M ZnSO_4_	~ 0.9	70.7%/400/1 A g^−1^	503 at 100 mA g^−1^	[85]
24	Zn^2+^	HATTA	Zn	1 M Zn(OTF)_2_	~ 0.6	84.07%/10000/25 A g^−1^	225.8 at 0.05 A g^−1^	[86]
25	Zn^2+^ and H^+^	DQDPD	Zn	1 M ZnSO_4_ + 2 M Na_2_SO_4_	0.8–0.9	92%/7500/10 A g^−1^	509 at 0.1 A g^−1^	[87]
26	Zn^2+^ and H^+^	BBQPH	Zn	3 M ZnSO_4_	~ 0.9	95%/1000/5 A g^−1^	498.6 at 0.2 A g^−1^	[88]
27	Zn^2+^ and H^+^	DNPT/rGO	Zn	3 M Zn(OTF)_2_	~ 0.8	94%/2000/3 A g^−1^	150 at 0.242 A g^−1^	[89]
28	Zn^2+^ and H^+^	TDT	Zn	1 M ZnSO_4_	0.78	81.3%/3000/10 A g^−1^	369 at 0.2 A g^−1^	[90]
29	Zn^2+^	TCNQ/CCP	Zn	1 M ZnSO_4_	~ 1.1	78.54%/100/1 A g^−1^	171 at 0.1 A g^−1^	[91]
30	Zn^2+^	TCNAQ	Zn	2 M ZnSO_4_	~ 1.2	81%/1000/500 mA g^−1^	166 at 50 mA g^−1^	[92]
31	Zn(OTF)^+^	p-DB hosted in carbon nanoflower	Zn	Zn(OTF)_2_ (pH = 3.29)	~ 0.7	93.9%/25000/5 A g^−1^	402 at 0.1 A g^−1^	[93]
32	Zn^2+^	TRT	Zn	2 M ZnSO_4_	~ 0.8	61.9%/1000/0.5 A g^−1^	170 at 0.1 A g^−1^	[94]
33	Zn(OTF)^−^	MB	Zn	3 M Zn(OTF)_2_	~ 0.8	86%/20000/16.7 A g^−1^	104.6 at 16.7 A g^−1^	[95]
34	Zn^2+^ and CF_3_SO_3_^−^	BDB	Zn	1 M ZnSO_4_-H_2_O and 2 M Zn(OTF)_2_-H_2_O	1.25	75%/1000/0.78 A g^−1^	112 at 0.39A g^−1^	[96]
35	Zn^2+^ and ClO_4_^−^	DMPZ	Zn	17 m NaClO_4_ and 0.5 m Zn(CF_3_SO_3_)_2_	1.45	0.5% decay per day/1000/255 mA g^−1^	231 at 51 mA g^−1^	[97]
36	TFSI^−^	dNPC	Zn	20 m LiTFSI + 1 m Zn(TFSI)_2_	1.3	96%/1000/0.5 A g^−1^	109 at 50 mA g^−1^	[98]
37	H^+^ and OTF^−^	PTDM	Zn	3 M Zn(OTF)_2_	1.13	65.6%/6400/1 A g^−1^	118.3 at 0.1 A g^−1^	[99]
38	ClO_4_^−^	TTF	Zn	10 m Zn(ClO_4_)_2_	~ 1.2	68.8%/10000/20C	215 at 2C	[100]

**Fig. 2:**
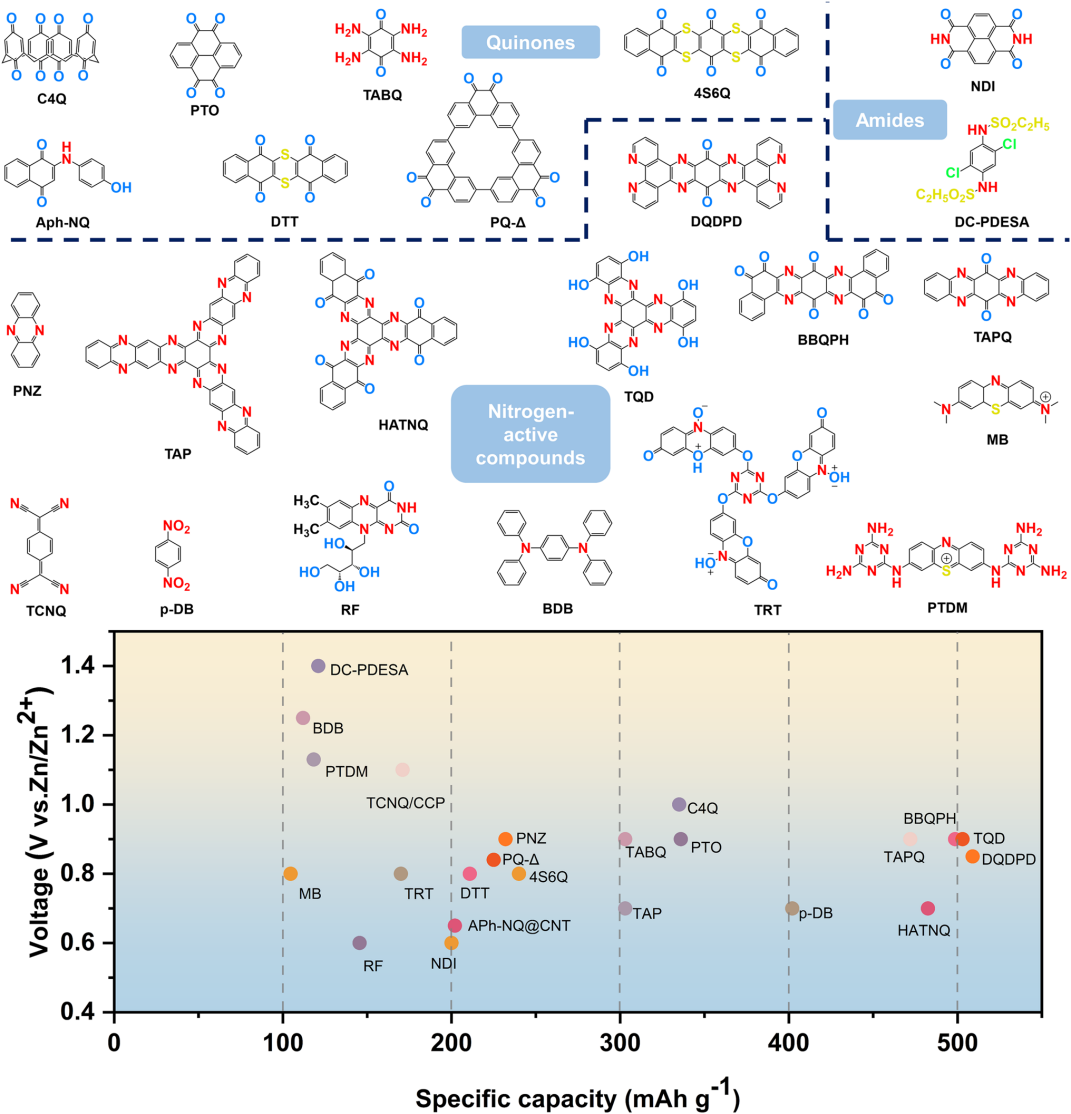
Molecular structures of representative organic small molecule electrode for AZIBs and comparison of their capacity and voltage

### Quinone-Based Derivatives

Quinone compounds readily dissolve in common ester and ether organic electrolytes because of the solubility principle, leading to a reduced proportion of active materials and severe battery capacity degradation. Relatively, quinone compounds have typically low solubility in water. As cathodes, the carbonyl group in quinone will be reduced during discharging, thus working as n-type materials binding with Zn^2+^ or H^+^. The utilization of quinone electrode materials in aqueous electrolytes can be dated back to 1972, when Sandstede et al. showed that tetrachlorobenzoquinone had a reduction potential of 0.7 V in a dilute H_2_SO_4_ solution [101]. In recent years, quinone-based materials have been extensively used in batteries, proving their stability across different pH levels, with various ions for current transport, over a wider temperature range, and in diverse atmospheres. Moreover, it can combine with other established cathode material to form a stable quinone-based aqueous battery [63].

**Fig. 3:**
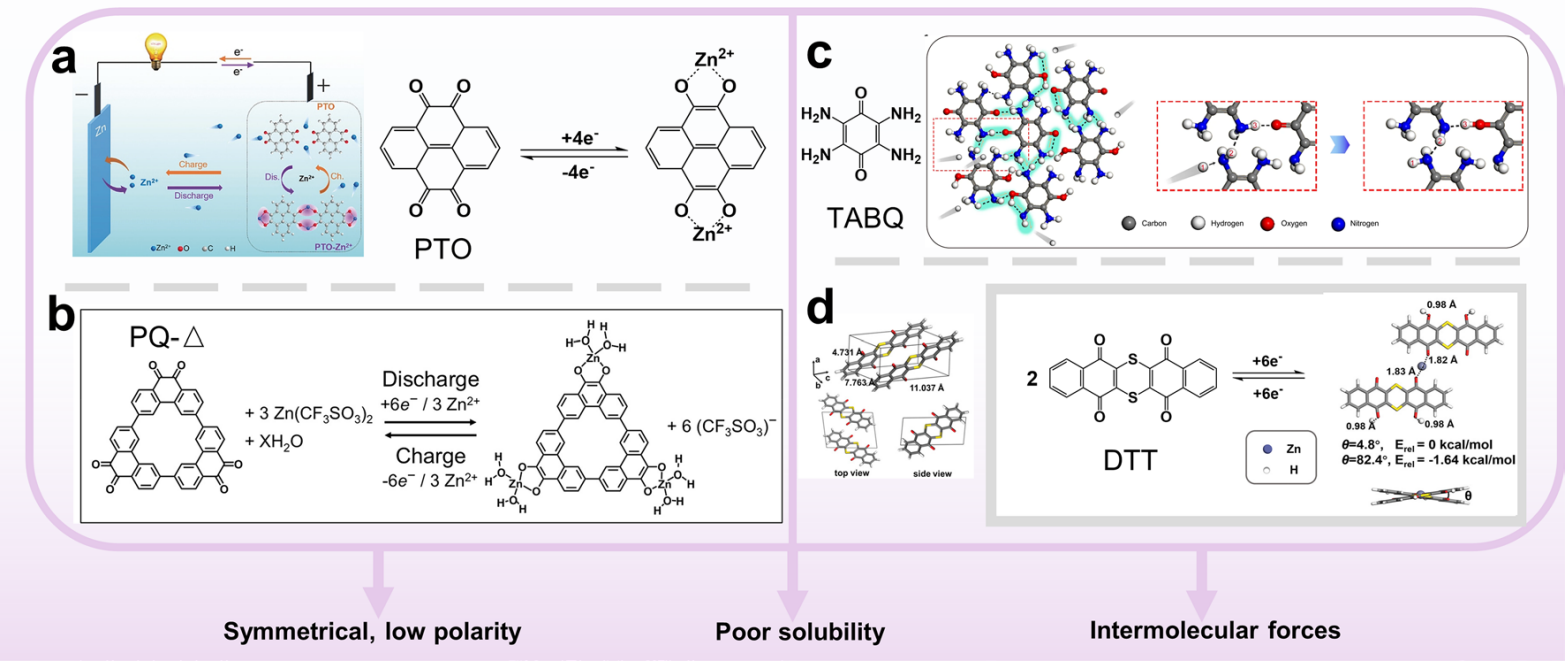
**a** Illustration of the reversible reaction mechanism of the aqueous PTO//Zn battery. Reprinted from Ref. [65] with permission from John Wiley and Sons. **b** Electrochemical redox chemistry of PQ-Δ. Reprinted from Ref. [67] with permission from American Chemical Society. **c** Schematic illustration for the proton conduction manner in the hydrogen bonding network in TABQ. Reprinted from Ref. [66] with permission under a Creative Commons Attribution 4.0 International License. **d** Crystal structure and optimized geometries and relative energies (E_rel_) of DTT_2_(H^+^)_4_(Zn^2+^). Reprinted from Ref. [68] with permission from John Wiley and Sons

In 2017, Yao's group first proposed the utilization of organic quinone compounds as a general anode material suitable for all kinds of aqueous rechargeable batteries [102]. The performance of aqueous metal-ion batteries was tested at different pH levels of electrolytes and different temperatures, employing organic compounds with 1,2-benzoquinone or 1,4-benzoquinone structures as anode and different metal oxides as cathode. They compared the performance parameters of the battery with the existing aqueous battery anode at that time and proved that the quinone material had stable performance, cheap price, and almost unlimited raw material resources. By optimizing the molecular structure design and cathode materials, the specific capacity of the battery is expected to be enhanced. Expanding on Yao's work, Chen's group conducted a comprehensive exploration of quinone electrodes in AZIBs for the first time [64]. Through the use of quinone cathode materials with featuring four benzene quinone units and eight carbonyl Calix, they were able to achieve a specific capacity of up to 335 mAh g^−1^ at a current density of 20 mA g^−1^, along with a high coulombic efficiency of 93% when employing a cationic-selective membrane. The capacity retention rate remained at 87% even after 1000 cycles at a current density of 500 mA g^−1^. Their research combined experimental findings with theoretical calculations, leading to the development of an electrostatic potential calculation method that demonstrated the key role of carbonyl groups in electrochemistry binding with cations. For the C4Q molecular structure, the carbonyls on and under a molecule are more favorable for Zn^2+^ uptake because they display lower ESP than the bilateral carbonyls. Furthermore, by utilizing in situ infrared spectroscopy, Raman spectroscopy, and UV–visible spectroscopy, they investigated the structural evolution and dissolution behavior of active materials during charge and discharge processes, thereby elucidating the mechanism behind reversible electrochemical reactions. Chen group's research highlighted the significance of the number and position of carbonyl groups in elucidating the mechanism. Shortly thereafter, wang's group discovered that a pyrene-4,5,9,10-tetraone (PTO) cathode with tetracarbonyl and planar conjugated structure was exceptionally well-suited for AZIBs (Fig. 3a) [65]. The presence of two carbonyl groups on the same side facilitated the chelation of divalent metal ions such as Zn^2+^. Subsequently, a variety of PTO-based materials including PTO-4NH_2_Ph [54], BT-PTO COF [56], and Tp-PTO-COF [103] were investigated, paving the way for a multitude of quinone-based electrodes in AZIBs.

However, the cycling stability will be affected by the gradual dissolution of quinone small molecules. Triangular small molecule quinones with rigid geometries are also have the potential to be low solubility materials [67]. The ring structure of PQ-Δ increases the electron delocalization and produces a complete six-electron reduced state, indicating enhanced stability. Furthermore, the DFT analysis showed that the insertion of H_2_O molecules and divalent ions synergistically reduced the desolvation energy (0.04 eV while 2.89 eV in organic one) and the coulomb repulsion on the electrode surface, suggesting improved reversibility and faster insertion/extraction efficiency. PQ-Δ in 3 M Zn(CF_3_SO_3_)_2_ exhibited a high capacity of 225 mAh g^−1^ and a coulomb efficiency of 99.6% at 30 mA g^−1^, suggesting that each PQ-Δ can accept three Zn^2+^ ions (Fig. 3b). Even at a rate of 150 mA g^−1^, the capacity remained 210 mAh g^−1^ after 500 cycles without obvious decay. The findings demonstrated that the cycling performance can be enhanced by the design of molecular structures possessing low polarity and expanded electron delocalization.

Slightly different from molecules with rigid structure, Sun et al. provided a strategy to this issue-introducing symmetric functional groups to confer molecular low dipoles to reduce the solubility of small molecules [66]. The utilization of tetraamino-p-benzoquinone (TABQ) as a cathode led to remarkable cycling stability and high rate performance, resulted from its high molecular symmetry and hydrogen bonding formed by amino and water molecules (Fig. 3c). Throughout the circulation process, alterations in the molecular charge characteristics often play a crucial role in influencing molecular solubility. In 2020, Wang et al. proposed the sulfur heterocyclic quinone dibenzo[b,i]thianthrene-5,7,12,14-tetraone (DTT) as a potential cathode [68]. The experimental and computational results showed that DTT can store both Zn^2+^ and H^+^. They gave possible structure of the discharge product–DTT_2_(H^+^)_4_(Zn^2+^), where two adjacent DTT molecules bind through a Zn^2+^ (Fig. 3d). Inherent insolubility of the bis-p-sulfide ring resists its solubilization during cycling. Apart from the optimization of organic structure, adopting an organic–inorganic hybrid cathode is also an effective option. Fu’s group proposed to restrict naphthoquinone-based small molecules in carbon nanotubes to reduce solubility [69], and through functional group modifications, such as dichlone and 2-((4-hydroxyphenyl) amino) naphthalene-1,4-dione (APh-NQ) have better cycle stability than anthraquinone, promoting the development of binder-free organic cathodes for AZIBs.

**Fig. 4:**
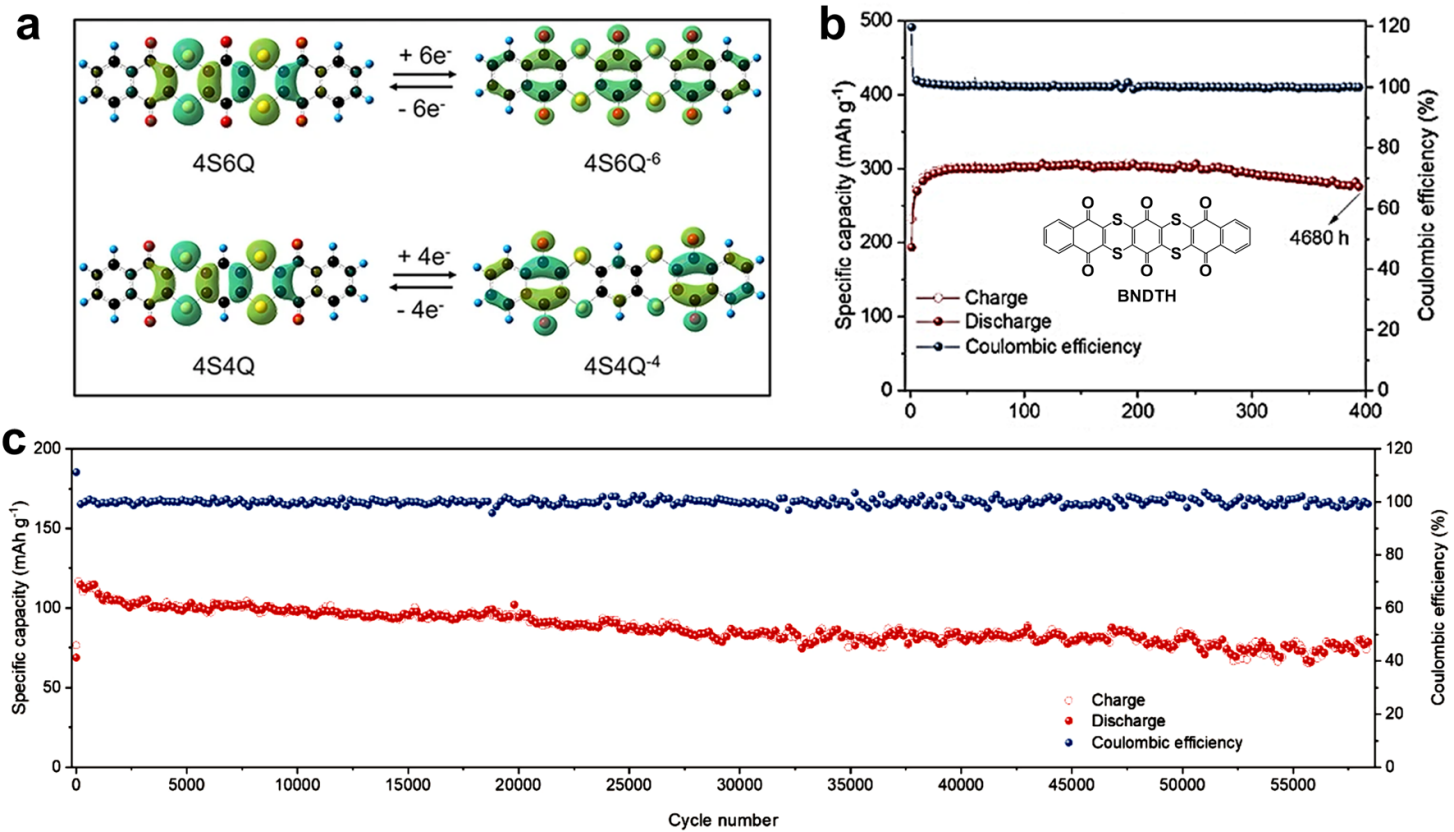
**a** Calculated HOMO plots of 4S4Q, 4S4Q^4−^, 4S6Q, and 4S6Q^6−^ molecules. Reprinted from Ref. [70] with permission under an open access Creative Common CC BY license. **b** Cycling performance of BNDTH/RGO at 0.05 A g^−1^. **c** Cycling performance of BNDTH/RGO at 10 A g^−1^. Reprinted from Ref. [104] with permission from John Wiley and Sons

In addition to the well-known concern about dissolution, scientists have also payed special attention to the inherent low electrical conductivity of organic materials. Recently, Tao's group introduced a new class of small sulfur heterocyclic quinones known as 4S4Q and 4S6Q [70]. These compounds not only show enhanced conductivity due to the inclusion of sulfur but also exhibit improved charge transfer capabilities owing to their antiaromatic ring structure. The DFT calculations demonstrated that all carbonyl groups can be reduced (Fig. 4a). Noteworthy, Zn^2+^ did not participate in the reaction of 4S6Q in 3.5 M Zn(ClO_4_)_2_ electrolyte through TEM mappings. However, Zhang's group prepared the same small molecule (called BNDTH) using solvent exchange, but it reflected the mechanism of storing Zn^2+^ in 2 M ZnSO_4_ that was distinct from Tao’s work [104]. The BNDTH electrodes combined with reduced graphene oxide exhibited superior redox-active site utilization, reaching a high capacity of 296 mAh g^−1^ and an extremely long cycling lifetime (58,000 cycles with a capacity retention of 65% at 10 A g^−1^) (Fig. 4b, c). These two works remind us that the identical molecular structure may exhibit distinct energy storage properties with different electrode preparation strategies or different electrolytes.

Therefore, the main challenge with redox-active quinone structures firstly remains in their solubility, which can be partially mitigated by creating symmetrical or inflexible molecules or incorporating insoluble elements. For the intrinsic poor conductivity, blending them with carbon materials is a practical way, which can deter dissolution and promote charge transfer, leading to improved overall electrochemical performances.

### Amide-Based Derivatives

Similarly, the electrochemical process of amides depends on the carbonyl active sites, which typically adopt the n-type mechanism. In contrast to quinones, the lone pair of electrons of nitrogen atom in amide-based derivatives enable boosted redox activity, improved resistance to hydrolysis, and novel ion storage approaches. The first use of amide materials for Zn^2+^ storage in aqueous systems was initiated by Li and coauthors [71]. They used 1,4,5,8-naphthalenetetracarboxylic dianhydride (NTCDI), 1,4,5,8-naphthalene diimide (NDI), and N, N′-diamino-1,4,5,8-naphthalenetetracarboxylic bisimide (DANTCBI) as cathodes for AZIBs, respectively. Experimental results showed that NTCDI has an incomplete carbonyl response due to potential spatial site resistance effects, and similar to DANTCBI, capacity decay rapidly. Among them, NDI had the best electrochemical performance with a capacity that was always above 200 mAh g^−1^. The X-ray photoelectron spectroscopy (XPS) results revealed that the insertion of Zn^2+^ deformed the NDI lamellar structure, which may lead to electron transfer or off-domain from the N site in the adjacent layer to the Zn site. To further maximize the capabilities of the NDI electrode, the group added Na_2_SO_4_ solution into the ZnSO_4_ electrolyte. Surprisingly, the initial discharge capacity in the electrolyte with 2 M Na_2_SO_4_ remained 157 mAh g^−1^ even after 5000 cycles, with a capacity retention of 76%, exceeding the 55% retention observed without Na_2_SO_4_ addition. Notably, Na^+^ effectively inhibited the growth of zinc dendrites, maintaining a smooth and flat zinc anode. Indeed, some imide-like structures exhibit the co-storage of Zn^2+^ with C=N and C=O. Affected by the biological metabolic process, a biomimetic riboflavin electrode material with an isooxypyrimidine ring was designed and synthesized by Wang’s group [73]. They not only proved that the isoxazine part is the active center (each pair of N and O cooperates to store 0.5 Zn^2+^), but also found the tetraoxazine and luminazine molecules obtained by cutting the molecular structure of riboflavin function as good cathode materials, which expands the direction of innovative organic electrode materials design.

To examine the impact of substituents on the cathode redox properties of organosulfonamides, three different substituents of N, N′-(1,4-phenyl)diazanesulfonamide (PDESA) compounds were synthesized as cathodes for AZIBs [74]. Due to the electron-withdrawing effect of Cl substituents on benzene rings, the N,N′-(2,5-dichloro-1,4-phenyl)diazanesulfonamide (DC-PDESA) cathode exhibited the highest battery voltage (~ 1.4 V) and the assembled AZIB demonstrated high open-circuit voltage (1.7 V), surpassing levels found in most aqueous zinc-ion batteries. Jin's group proposed to assemble aqueous zinc-ion batteries using cyclodextrin-based volumetric effect electrolyte and organic conjugated sulfonamide cathode material at the same time, which significantly improved the operating voltage, cycle stability, and operating temperature range of AZIBs [105]. To illustrate the improvement in electrolyte performance through the exclusion volume effect of macromolecules, various concentrations of highly water-soluble, electrochemically inert, and cost-effective (2-hydroxypropyl)-β-cyclodextrin (HBCD) supramolecules were added to the Zn(CF_3_SO_3_)_2_-based electrolyte. HBCD possessed a molecular size approximately two orders of magnitude larger than H_2_O, resulting in a significant exclusion volume effect within the electrolyte. Additionally, HBCD featured numerous hydroxyl side groups, promoting the formation of an extensive hydrogen bond network with water molecules. The combined impact of the volume effect and the hydrogen bond network substantially diminished the reactivity of water molecules and broaden the electrolyte's electrochemical window.

Overall, the scarcity of amide-based small molecules in aqueous systems can be attributed to two main reasons: (1) most amide-derivatives rely on carbonyl groups for energy storage, which is similar to quinones, and (2) polyimide have been widely used in aqueous batteries due to their superior designability and cycling stability than small molecules. Nevertheless, the presence of electron-rich N-atoms still lays the foundation for efficient energy storage, which has inspired the exploration of new structures, as discussed in the following section.

### Nitrogen-Active Compounds

**Fig. 5:**
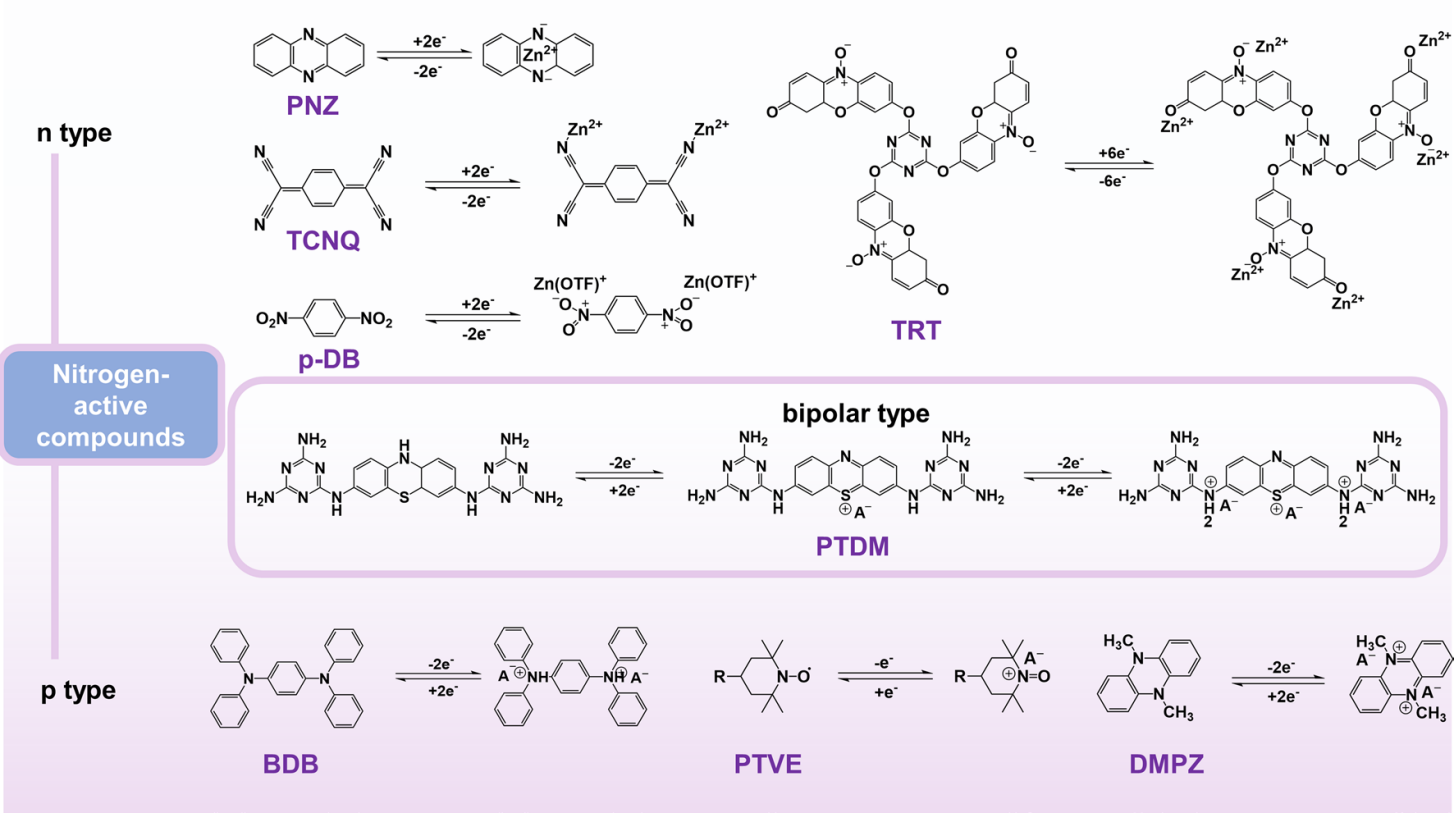
Energy storage mechanism of several nitrogen-active compounds

Nitrogen-active compounds have been extensively exploited as the electrochemical active materials due to their rich storage sites and large insoluble frameworks. It is well known that N could not only improve the electronic conductivity, but also increase the redox potential due to the fact that its electronegativity is between that of C and O. Apart from nitrogen-containing conductive polymers and nitroxide radicals, nitrogen-active compounds have shown more and more significant application prospects in AZIBs in recent years. Herein, various nitrogen-active small molecules have been discussed, including linear/tridentate structure with C=N bond, conjugated aromatic ring with tetracyano, azo compounds and integrated multiredox centers with C=N and C=O bonds, all of which involve N/P-type materials and radical chemistry (Fig. 5).

Phenazine (PNZ) and its analogues have been demonstrated to be effective electrode materials based on their redox-active imino group (C=N), in which electron-rich N atom allows C=N bond to adsorb Zn^2+^. For instance, 2,3-diaminophenazine (DAP) was served as a cathode material in AZIBs [75]. While the robust π–π interaction provides DAP with electronic conductivity and structural stability, the presence of hydrophilic amino group can expedite DAP's dissolution in aqueous electrolytes due to the likelihood of hydration through the formation of hydrogen bonds. The CNT-modified separator was used to inhibit the dissolution of DAP, thus achieving high-rate (500C) and long-cycle (10,000 cycles) performance. Compared with DAP, there are no hydrophilic groups and hydration on its peripheral positions for PNZ, creating long-cycle life with 79% of initial capacity after 1,000 cycles without modification of separator.

In addition to linear structure, tridentate PNZs have remarkable advantages including multi-active centers and extended π-conjugated system. As an illustration, diquinoxalino[2,3-a:2′,3′-c]phenazine (DQP) molecules were designed and displayed an ultrahigh capacity of 413 mAh g^−1^ at 50 mA g^−1^ as the cathode material for AZIBs [76]. Furthermore, HATN, as a typical cathode, realized the insertion/removal of protons in the aqueous organic battery, resulting in a discharge capacity of 405 mAh g^−1^ in the first cycle [77]. After that, the electrode research based on HATN in AZIBs has gradually deepened.

**Fig. 6:**
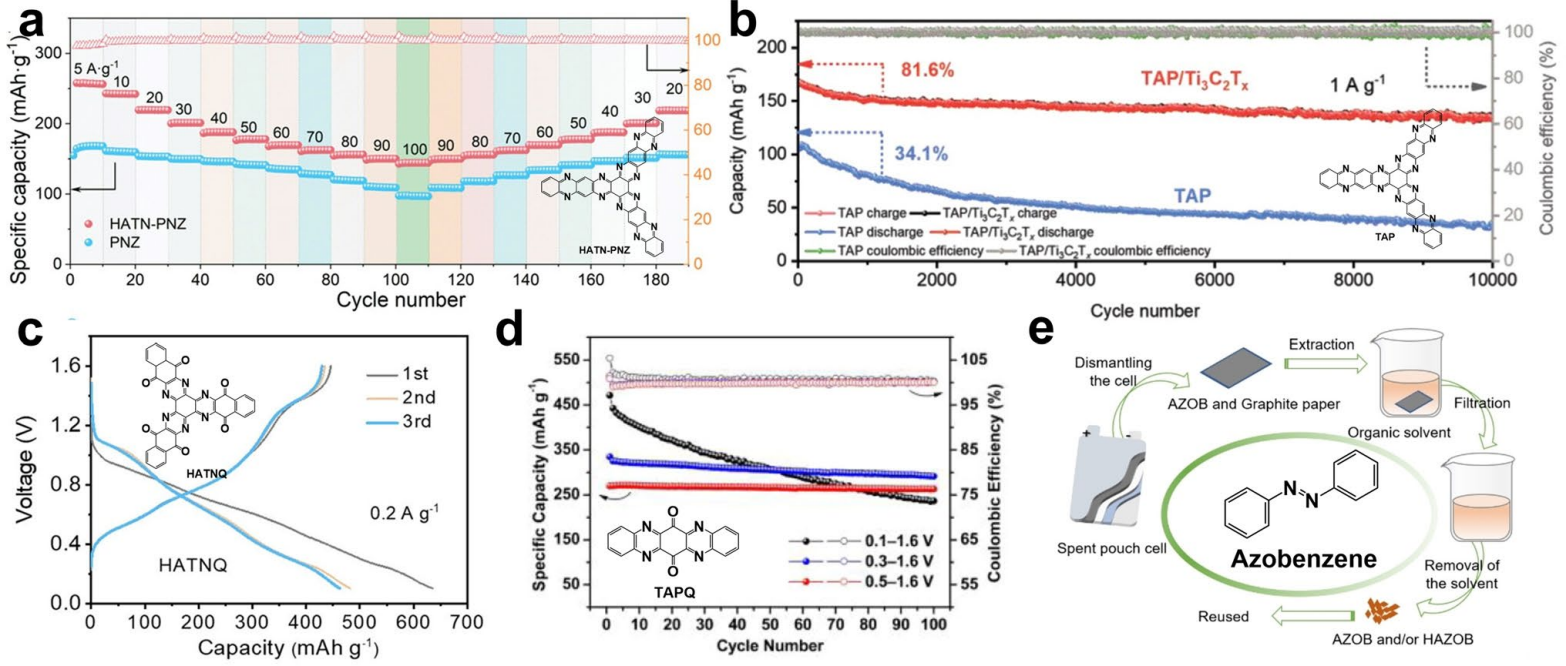
**a** Rate specific capacity of HATN-PNZ and PNZ. Reprinted from Ref. [79] with permission from John Wiley and Sons. **b** Long-term cycling test of TAP and TAP/Ti_3_C_2_T_x_ at 1.0 A g^−1^. Reprinted from Ref. [80] with permission from John Wiley and Sons. **c** Galvanostatic discharge/charge curves of the HATNQ electrodes at 0.2 A g^−1^. Reprinted from Ref. [81] with permission from John Wiley and Sons. **d** Cycling performance of TAPQ at 50 mA g^−1^. Reprinted from Ref. [84] with permission from Elsevier Ltd. **e** Schematic diagram of the regeneration process. Reprinted from Ref. [106] with permission from John Wiley and Sons

Incorporating a cyano group (-CN) into the conjugated HATN is a novel and efficient approach. The presence of electron-withdrawing groups lowers the LUMO energy, thus increasing the operating voltage [78]. Recently, Cheng's group prepared π electron-conjugated nitrogen-heteroaromatic ring organic material (HATN-PNZ) by dehydration condensation reaction [79]. The electron delocalization area can be effectively increased by the extension of π-conjugated aromatic ring, resulting in reduced energy difference between HOMO and LUMO and improved electron transport efficiency within the molecule. Moreover, the polymerization can inhibit the dissolution of organic materials in electrolytes; thus, the batteries with HATN-PNZ cathode exhibited an exceptionally high-rate performance (Fig. 6a) and ultra-long-cycle life.

It is well known that the redox pseudocapacitance dominant phenomenon and Zn^2+^/H^+^ co-storage mechanism of the battery usually result in excellent power density and energy density. Huang's group proposed an imine-based tris(aza)pentacene (TAP) with extended conjugation effects along the C=N backbones [80]. It is injected in situ into layered Mxene to form the TAP/Ti_3_C_2_T_x_ cathode. Theoretical and electrochemical analysis revealed the selective H^+^/Zn^2+^ co-insertion/extraction mechanism of TAP, which is attributed to the effect of spatial site effects on the validity of the active C=N site. Notably, Ti_3_C_2_T_x_ as a conductive scaffold facilitates the rapid diffusion of Zn^2+^ and enhance the TAP electrode kinetics. The close electron interaction between TAP and Ti_3_C_2_T_x_ ensured the structural integrity of TAP/Ti_3_C_2_T_x_ during repeated charge and discharge. Thus, the TAP/Ti_3_C_2_T_x_ cathode provided a high reversible capacity of 303 mAh g^−1^ at 0.04 A g^−1^ and achieved an ultra-long lifetime of more than 10,000 cycles with a capacity retention of 81.6% (Fig. 6b).

An alternate strategy to achieve high energy density is exploiting potential functions of hydrogen bonds and introduce denser active sites. Wang's research revealed that hexaazatrinaphthalene-quione (HATNQ) has strong polarity due to the existed many C=O and C=N bonds, resulting in a large number of hydrogen bonds between adjacent molecules and the formation of a two-dimensional layer structure [81]. The combination of the high density of hydrogen bonds and the inherent π–π conjugation system synergistically enhanced ion transport and insolubility, enabling the electrode to deliver a high capacity of 482.5 mAh g^−1^ at a current density of 0.2 A g^−1^ (Fig. 6c). In order to reduce the dead mass of HATNQ, Song's group designed and synthesized a simpler structure of 5,7,12,14-tetranitrogen-6,13-pentaquinone (TAPQ) [84], which reached a specific capacity of 443 mAh g^−1^ at 0.05 A g^−1^ (Fig. 6d). More importantly is that they revealed for the first time that the main reason for the decay of TAPQ capacity is the deterioration of the electrode structure caused by the change of the crystal structure and the residual accumulation of Zn_4_SO_4_(OH)_6_·5H_2_O by-products, rather than the dissolution of active material as previously generally thought. Similar work has been done with the recently published TQD molecule, which subtracts the part of the benzene ring from HATNQ. Surprisingly, the band gap became narrower and the theoretical capacity increased to 669 mAh g^−1^, indicating to a 12-electron reaction mechanism [85].

In addition to the above HAT structure, researchers have been gradually recognizing another extended nitrogen-containing heterocyclic conjugation system, with intrinsic insolubility and high conductivity during cycle. Through the utilization of schiff base reaction, a new nitrogenous heterocyclic super-conjugated organic compound bipyridine[3′,2′:5,6;2″,3″:7,8]quinoxaline[2,3-i]bipyridine[3,2-a:2′,3′-c]benzizine-10,21-dione (DQDPD) was synthesized [87]. DQDPD not only provided a record reversible specific capacity with 509 mA g^−1^ at 0.1 A g^−1^, but also exhibited excellent cycling performance (92% capacity retention after 7500 cycles at 10 A g^−1^) and rate performance (161 mAh g^−1^ at ultra-high current density of 20 A g^−1^). Liu et al. proposed the superelectron delocalization structure molecule BBQPH. The introduction of additional carbonyl groups effectively reduced the HOMO level and greatly narrowed the band gap. Thus, it showed excellent rate performance while maintaining a specific capacity of 393.6 mAh g^−1^ at 8 A g^−1^ [88].

Cyano can also be used to coordinate with Zn^2+^. Nagarale's group reported a small-molecule 7,7,8,8-tetracyanoquino dimethane (TCNQ) electrode [91]. The characterization revealed a correlation between the charge/discharge process and the formation/deformation of Zn-TCNQ complexes. To inhibit dissolution of complexes, they limited TCNQ to newly prepared covalent organic polymer CCP, leading to improved capacity and stability. Shen's group stabilized the crystal structure of TCNQ and reduced its solubility by adding high-valent Al^3+^ cations to the Na_2_SO_4_/ZnSO_4_ aqueous electrolyte [107], which not only prevented the solubilization of TCNQ but also increased specific capacity and cycle life owing to the embedding/deintercalation of high-valent cations (Al^3+^ and Zn^2+^) in TCNQ that was highly reversible. By further increasing the structure of TCNQ, π–π-conjugated system yielded the structure of tetracyanoanthraquinodimethane (TCNAQ) [92]. Through simulation calculations and characterization, Yao's group proved that the cyanide group can bond to Zn^2+^ during the charging and discharging process, thereby expanding the voltage window of the battery to 1.8 V.

Recently, the azo site has been found to bind with H^+^ [106]. Wang's group successfully developed the azobenzene cathode, which could bind two H^+^ when discharged and remove H^+^ when charged. In addition, it also showed excellent capacity and stability under high mass loading. More importantly, thanks to the redox stability, the electrode can be recycled in any state of charge and discharge (yield above 90%) (Fig. 6e), paving the way for practical applications.

Apart from the above-mentioned active functional groups, nitro groups also have redox activity [93]. Theoretical calculations revealed the important influence of nitro structural isomerization on the aromatic backbone, which changed the intramolecular electron distribution and host energy level, resulting in different zinc-loving activities and redox kinetics. The two-step redox reaction of cationic Zn(OTF)^+^ complexes in nitroaromatic cathodes was elucidated. Completely different from alkali metal-ion batteries, this charge storage mechanism prevented nitroaromatic hydrocarbons from rearranging their configuration and sharing Zn^2+^ with adjacent nitroelements, thereby reducing desolvation energy loss and maintaining structural stability in electrochemical reactions. Benefiting from composite strategy, p-DB encapsulated in carbon nanoflowers (p-DB@CF) as organic cathode provided a large reversible capacity of 402 mAh g^−1^ and utmost stability up to 25,000 cycles, which made batteries a high energy density of 230 Wh kg^−1^. Further, the electrochemical indexes of nitroaromatic hydrocarbons can be controlled by adjusting the side groups with electron-withdrawing/pushing functional substituents on the nitrobenzene backbone.

Nitroxy radical electrodes are also developing rapidly. Gaubicher's team used the nitroxide radical derivative 4HT-Benzene as the cathode and the imide as the anode to form an all-organic battery [108]. 4HT-Benzene undergoes a one-electron oxidation process to produce the oxoammonium cation, the charge being counterbalanced by the uptake of one anion from the electrolyte. Liu et al. prepared conjugated TRT molecule by retaining carbonyl and nitroxide radical in Resazurin sodium salt as active sites. The nitrogen-rich characteristics of triazine and the new n-type nitroxide radical achieved reversible storage of six Zn^2+^. The band gap of the whole molecule was narrowed, which is a feasible direction for future structural design [94]. Except for free radical compounds, people are becoming aware of the high activity of free radical intermediates in the exploration of ultrafast zinc batteries. Using methylene blue (MB) as cathode, Huang et al. found that it had a high electron transfer rate constant of 0.32 cm s^−1^ and retained the original 40.7% capacity as the current density increased from 1 to 500C (83.5 A g^−1^) [95]. They demonstrated via the EPR signal that MB molecules undergo free radical intermediates when binding to Zn^2+^, which implied a reduction in the reaction energy barrier. These efforts broadened the path of free radical chemistry in AZIBs.

**Fig. 7:**
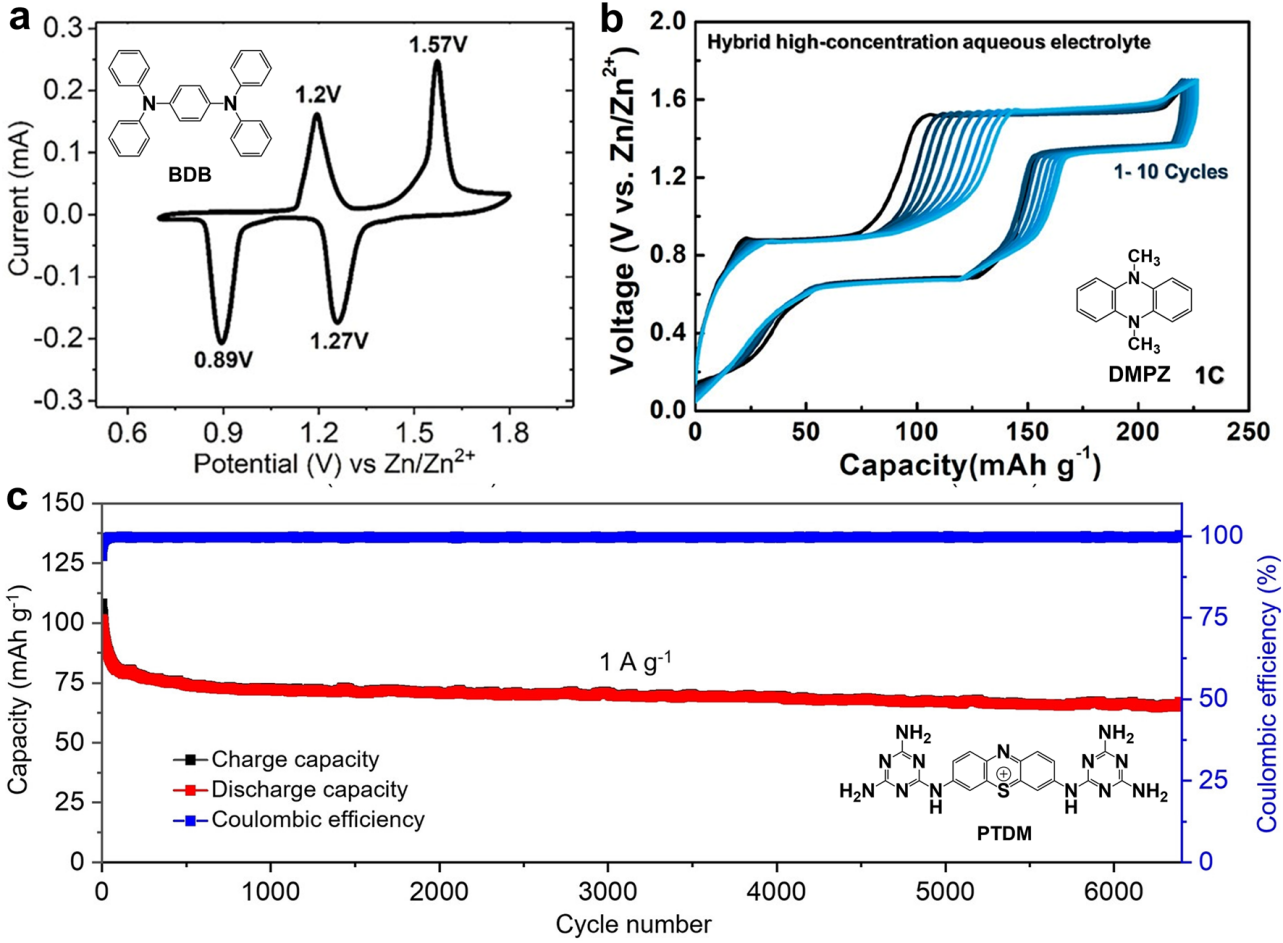
**a** Cyclic voltammogram at a scan rate of 0.1 mV S^–1^. Reprinted from Ref. [96] with permission from American Chemical Society. **b** Charge–discharge curves of the DMPZ electrode for the initial 10 cycles. Reprinted from Ref. [97] with permission from American Chemical Society. **c** Cycling performance of the PTDM//Zn battery. Reprinted from Ref. [99] with permission from John Wiley and Sons

Batteries based on nitroxide radicals often face the challenge of a narrow voltage window due to the instability of the radicals at high voltages. The use of p-type materials with embedded anions is considered to be an effective way to increase voltage, such as the typical trianiline small molecule—1, 4-diphenylbenzene (BDB) [96]. CV curves and operando XRD showed that the reaction of BDB has two steps (Fig. 7a), with an intermediate BDB^+^·A^−^ generated, and the binding site is the nitrogen cation. One thing to note is that although neutral BDB is insoluble, its oxidized forms BDB^+^·A^−^/BDB^2+^·A^2−^ are partially soluble in highly concentrated electrolytes, which was solved by using cellulose nanocrystal membrane (CNC). Similarly, 5,10-dihydro-5,10- dimethylphenazine (DMPZ) also underwent a two-electron redox process to generate DMPZ^+^, DMPZ^2+^ with embedded anions (Fig. 7b) [97]. Considering the compatibility of electrode/electrolyte, researchers predicted and designed a high-concentration mixed electrolyte with 17 m NaClO_4_. The experiment proved that DMPZ showed excellent cycling performance (a cycle degradation rate of below 0.5% per day over 1000 cycles at 1C and 5000 cycles at 5C). A recent report shows the p-type energy storage capacity of small aniline-like molecules. The researchers cleverly exploited the bipolarity of phenothiazine and linked it to the nitrogen-rich triazine ring, named PTDM. As its linking fragment, -NH- fully demonstrated the possibility of anion storage and showed stable cycling performance (Fig. 7c) [99]. These studies indicated the usefulness of p-type/bipolar electrodes in aqueous batteries.

**Fig. 8:**
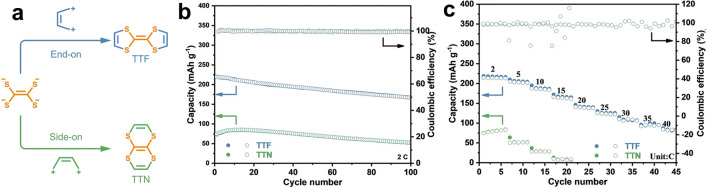
**a** Schematic of two isomers TTF and TTN. **b** Cycling performance of TTF and TTN electrode at 2 C. **c** Rate capability of the TTF and TTN electrode. Reprinted from Ref. [100] with permission under an open access Creative Common CC BY license

In addition to the specific N-containing active structures of the p-type electrode, small molecules containing S also show impressive electrochemical properties. Wang et al*.* reported two small molecules (TTF and TTN) that are isomers as cathodes for AZIBs (Fig. 8a) [100]. They found that TTF could store two monovalent anions reversibly, while TTN could store only one. This indicates that the same sites in different structurally isomerized molecules will have different electrochemical behaviors. At low current density, TTF exhibited a capacity of 215 mAh g^−1^, while maintaining an excellent capacity of 81 mAh g^−1^ at 40C, both better than that of TTN (Fig. 8b, c). In addition, TTN was found to undergo irreversible molecular rearrangement to become TTF as the voltage widens to 2.2 V.

It should be noted that there are different mechanisms of cation and anion storage due to the difference of N active sites and other linked segments. Current research is mainly focused on capacity and cycling stability, and still new nitrogen-containing active sites are being explored. It is also believed that the electronic conductivity and insolubility can be enhanced by building larger conjugated structures. At the same time, the electronic nature of the fragments needs to be taken into account when introducing bipolar structures. Generally, the energy gap of D-A structures is narrower, which facilitates electron transport and is beneficial to the rate performance of batteries. With the proceeding of such research, nitrogen-active molecules are expected to be a breakthrough for ultra-high performance in AZIBs.

In essence, the clarity and adaptable structural design of redox-active organic small molecules prove instrumental in probing the intricate relationship between their structure, functional groups, and electrochemical performance. More importantly, the development of AZIBs cannot be off-track from practical applications. In this concern, the advantages of low synthesis cost and ease of mass production for small molecules are of great significance. However, these molecules also present challenges such as low conductivity, high solubility, and instability under elevated voltages. Despite the fact that refining their structure or incorporating inorganic material hybrids can enhance their electrochemical characteristics, this invariably extends the complexity of synthesis and amplifies costs. Therefore, employing polymerization reactions to transform small molecules into meticulously organized polymers becomes a highly promising strategy. This method not only resolves major concerns associated with sever dissolution and voltage thresholds, but also raise the possibility of elevating the conductivity.

## Non-porous Polymers Enable Stable Electrodes

**Fig. 9:**
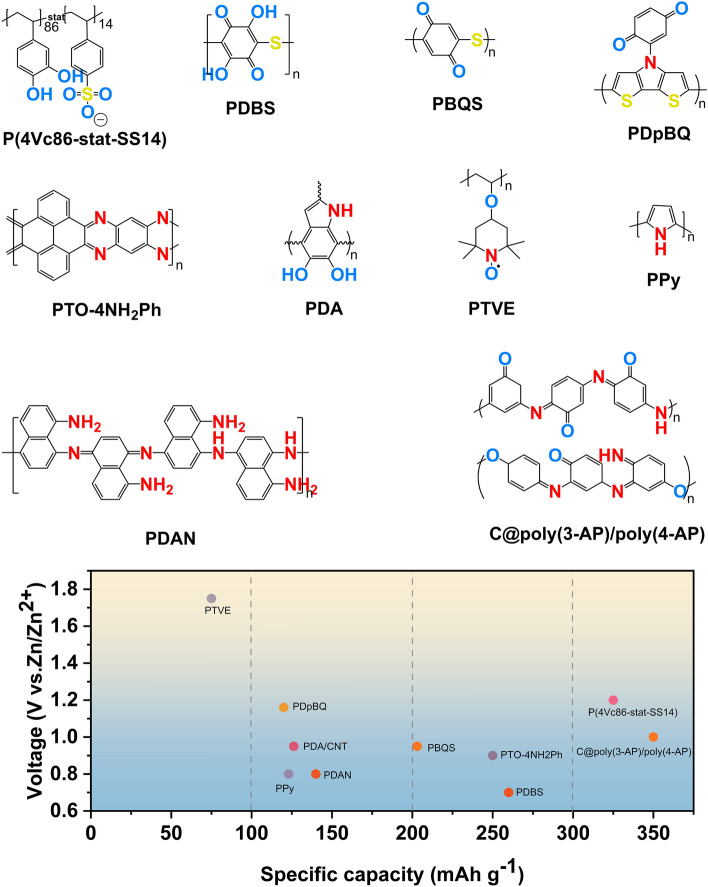
Molecular structures of representative linear polymer electrode for AZIBs and comparison of their capacity and voltage

The aforementioned dissolution problem of redox-active organic small molecules presents a significant challenge toward the practical application of AZIBs. The utilization of composite strategies frequently leads to a diminished specific capacity. In this regard, polymers stand out due to their poor solubility in electrolytes, tunable specific capacity, crystallinity, processability, and mechanical properties. In this section, we categorize non-porous polymers according to their redox active centers into quinone-based, aniline-based, and nitrogen-active heterocyclic linear polymers. The representative examples are also summarized to exhibit their electrochemical performances (Fig. 9 and Table 2).

**Table 2:** Summary of electrochemical properties of linear polymer

#	Charge carriers	Cathode	Anode	Electrolyte	Output voltage (V)	Cycling stability	Specific capacity(mAh g^−1^)	Refs
1	Zn^2+^	P(4VC_100_)	Zn	4 M Zn(TFSI)_2_	1.2	–	350 at 0.35 A g^−1^	[109]
2	Zn^2+^	P(4VC_86_-stat-SS_14_)	Zn	4 M Zn(TFSI)_2_	1.2	74%/400/0.35 A g^−1^	325 at 0.35 A g^−1^	[109]
3	Zn^2+^	PDBS	Zn	2 M ZnSO_4_	0.7	79%/2000/2 A g^−1^	260 at 0.01 A g^−1^	[110]
4	Zn^2+^	PBQS	Zn	3 M Zn(CF_3_SO_3_)_2_	0.95	86%/50/40 mA g^−1^	203 at 20 mA g^−1^	[111]
5	Zn^2+^	PPPA	Zn	2 M Zn(CF_3_SO_3_)_2_	~ 0.7	70.6%/20000/5 A g^−1^	210 at 50 mA g^−1^	[112]
6	Zn^2+^ and CF_3_SO_3_^−^	PONEA/GO	Zn	3 M Zn(CF_3_SO_3_)_2_	~ 0.8	85%/4800/10 A g^−1^	329 at 0.1 A g^−1^	[113]
7	Zn^2+^	PDpBQ	Zn	2 M ZnSO_4_	1.16	79%/500/2 A g^−1^	120 at 0.1 A g^−1^	[114]
8	Zn^2+^ and H^+^	OAP	Zn	3 M Zn(CF_3_SO_3_)_2_	~ 0.5	85.8%/500/0.1 A g^−1^	129.6 at 0.2 A g^−1^	[115]
9	Zn^2+^	PDA/CNT	Zn	3.3 M ZnSO_4_	0.9–1.0	96%/500/200 mA g^−1^	126.2 at 20 mA g^−1^	[116]
10	Zn^2+^ and Cl^−^	PANI	Zn	2 M ZnCl_2_ and 3 M	–	Nearly constant/1000/8 A g^−1^	203.5 at 0.5 A g^−1^	[117]
				NH_4_Cl	0.8			
10	Zn^2+^ and Cl^−^	PANI@celloluse yarn	Zn	2 M ZnCl_2_/3 M NH_4_Cl gel	1.0–1.2	91.9%/1000/5 A g^−1^	189.1 at 0.2 A g^−1^	[118]
11	SO_4_^2−^	Q-PANI	Zn	1 M ZnSO_4_	1.1–1.2	88.0%/1500/2 A g^−1^	186 at 0.2 A g^−1^	[119]
12	Zn^2+^ and CF_3_SO_3_^−^	PANI	Zn	1 M Zn(CF_3_SO_3_)_2_	1.1	92%/3000/5 A g^−1^	200 at 0.05 A g^−1^	[120]
13	Zn^2+^, H^+^ and SO_4_^2−^	PANI-S	Zn	1 M ZnSO_4_	1.1–1.2	70%/200/2 A g^−1^	180 at 2 A g^−1^	[121]
14	Cl^−^	PANI	Zn	2 M ZnCl_2_ and 3 M NH_4_Cl	1.09	97.5%/150/2.5 mA cm^−2^	133.9 at 2.5 mA cm^−2^	[122]
15	Zn^2+^ and CF_3_SO_3_^−^	PDAN	Zn	0.5 M Zn(CF_3_SO_3_)_2_	~ 0.8	Keep constant/1000 cycles/2 A g^−1^	140 at 0.1 A g^−1^	[123]
16	H^+^	PTO-4NH_2_Ph polymer	Zn	3 M ZnSO_4_	~ 0.9	83%/5000/5 A g^−1^	250 at 0.5 C (1C = 0.25 A g^−1^)	[54]
17	H^+^	PO	PR	2 M ZnSO_4_	~ 0.6	94%/500/1 A g^−1^	147 at 0.1 A g^−1^	[115]
18	Zn^2+^	C@poly(3-AP)/poly(4-AP)	Zn	2 M ZnSO_4_	1.0	91%/2000/5 A g^−1^	350 at 0.2 A g^−1^	[124]
19	Zn^2+^ and Cl^−^	PTVE	Zn	0.1 M ZnCl_2_ and 0.1 M NH_4_Cl	1.73	65%/500/60C	131 at 45 μA cm^−2^	[125]
20	Zn^2+^ and Cl^−^	PPy	Zn	PVA–KCl–Zn(CH_3_COO)_2_ gel	~ 0.8	38%/200/4.4 A g^−1^	123 at 1.9 A g^−1^	[126]

### Quinone-Based Polymer

The carbonyl group plays a vital role in quinones by providing a high specific capacity as the primary redox-active site. However, some quinones tend to form hydrogen bonds with water in their discharge products, leading to reduced utility of active sites. Moreover, as free monomers, organic small molecules contribute limited electrons and ions transfer, resulting in low conductivity. Polymerizing small molecules into linear polymers has emerged as an effective approach. This strategy not only reduces solubility by increasing the molecular weight, but can also introduce side groups or extend the conjugated structure to enhance charge transport within the polymer, thus accelerating electron transport and significantly improving the conductivity.

**Fig. 10:**
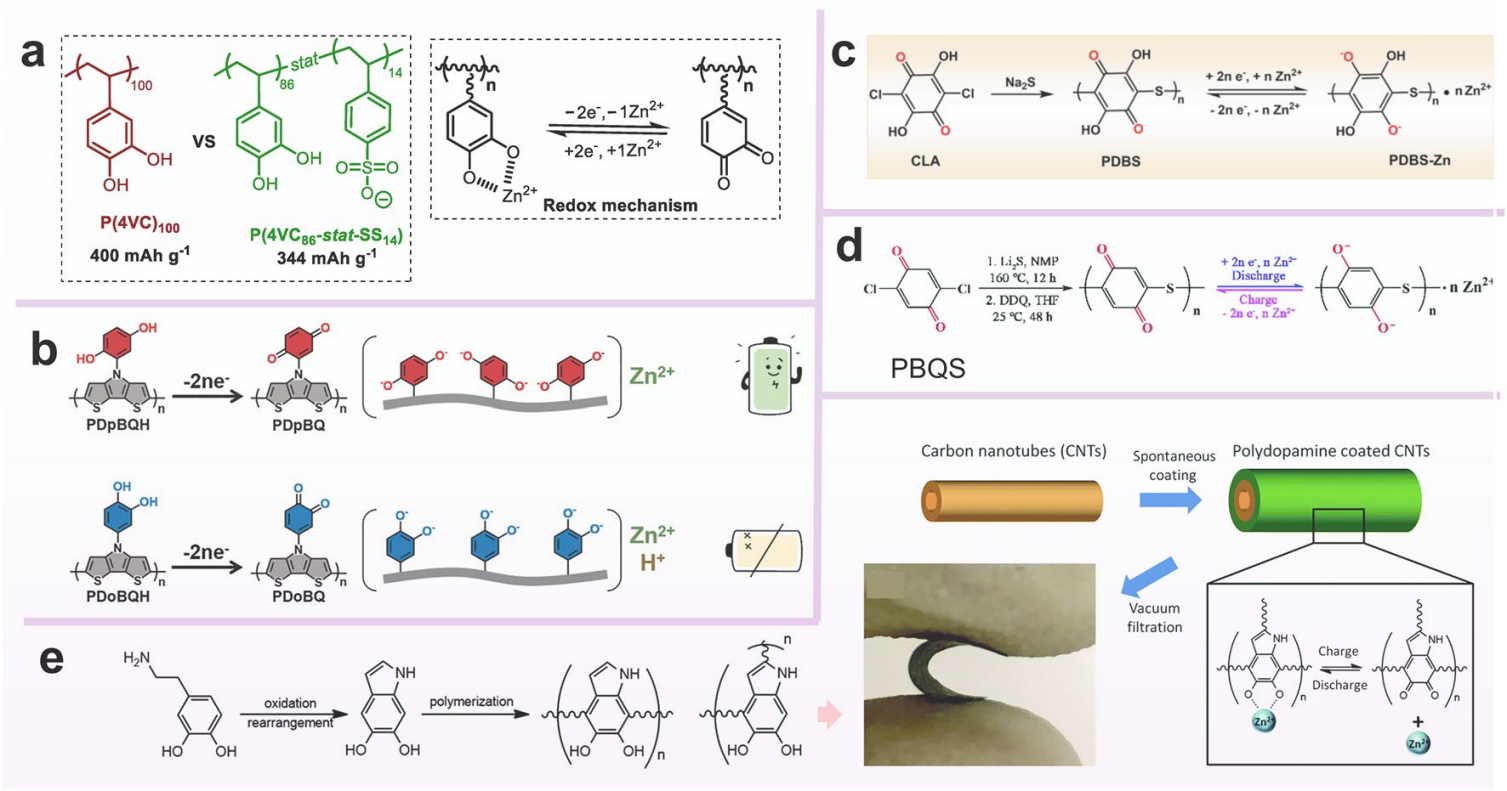
**a** Chemical structure of poly(catechol) homo and copolymers, along with simplified redox mechanism. Reprinted from Ref. [109] with permission under an open access Creative Common CC BY license. **b** Schematic of PDpBQH/PDoBQH were oxidized to work in ZIBs. Reprinted from Ref. [114] with permission from John Wiley and Sons. **c** Preparation of PDBS and proposed redox mechanism for ZIBs. Reprinted from Ref. [110] with permission from John Wiley and Sons. **d** Synthetic route and the proposed electrochemical redox mechanism of PBQS. Reprinted from Ref. [111] with permission from Royal Society of Chemistry. **e** PDA/CNTs electrode and its energy storage diagram. Reprinted from Ref. [116] with permission from Royal Society of Chemistry

To gain better control over the impact of functional groups on electrochemical performance, scientists conducted analyses from both the perspective of polymer structure and conduction mechanisms. According to previous studies on monovalent ion batteries conducted by Rebeca’s group, the incorporation of anionic copolymer monomers into the polymer structure effectively enhances the battery performance [127, 128]. In order to extend the research to multivalent ion batteries in aqueous systems, Rebeca’s group synthesized catechol homopolymers (P(4VC)_100_), catechol and sodium styrene sulfonate copolymers (P(4VC_86_-stat-SS_14_)) for cathode material, respectively (Fig. 10a) [109]. With increasing C-rate, the capacity of the P(4VC_86_-stat-SS_14_) copolymer showed a significantly smaller decrease compared to the P(4VC)_100_ homopolymer. This suggests that P(4VC_86_-stat-SS_14_) copolymers exhibit superior rate performance when discharged. Furthermore, the discharge capacity of the P(4VC_86_-stat-SS_14_) copolymer consistently outperforms that of the P(4VC)_100_ homopolymer at temperatures below 0 °C. These tests demonstrate that the addition of conjugated conducting anionic monomer pendants, such as sulfonates, to the polymer chains leads to a notable improvement in rate performance, low-temperature performance, and overall capacity. This improvement is attributed to enhanced Zn^2+^ mobility facilitated by a lower energy barrier in the redox reaction involving sulfonates. Nonetheless, the conductivity of the main chain also needs to be considered. Tang's research group suggested the utilization of a bis-thiophene polymerized with pyrrole units to create a polymer backbone, ensuring both high electron transport and structural stability. They incorporated hydroquinone and pyrocatechol as side groups, naming the resulting polymers PDpBQ and PDoBQ, respectively [114]. It was determined through DFT calculations that both PDpBQ and PDoBQ exhibited twisting at the N position. Furthermore, upon the oxidation of the quinone group, the LUMO level decreased, resulting in a narrower energy band gap. CV curves in AZIBs demonstrated that PDpBQ exclusively coordinated with Zn^2+^, while PDoBQ also exhibited reactivity with H^+^ (Fig. 10b). However, PDoBQ exhibited lower energy storage performance, and there was a 45% capacity degradation within the initial 5 GCD cycles. Ultimately, pBQZn-2 is considered to be the most probable binding form of PDpBQ to carriers due to its lower energy band gap (1.88 V) and more uniform electron delocalization. As for PDoBQ, it presents a wider energy band gap (2.66 V) and experiences high resistance in the vertical direction of the primary backbone.

Thioether structures have been extensively incorporated into quinone polymers. Thioether structures, in contrast to the low energy barrier of salt ions, tend to alter the polymer's steric morphology, thereby facilitating the intercalation and deintercalation of ions. Additionally, thioether structures play a crucial role in decreasing the solubility of quinones and improving cycling stability. Based on this, Zhang’s group designed poly(2,5-dihydroxy-1,4-benzoquinonyl sulfide) (PDBS) as cathode [110]. The experimental results demonstrated that the unique helical and foldable structure, along with the flexible lattice of the polymer, promoted the insertion and extraction of Zn^2+^ (Fig. 10c). Meanwhile, both O and S can be used as coordination sites for Zn^2+^; therefore, the battery presented a capacity of up to 260 mAh g^−1^ at 0.01 A g^−1^ and excellent cycle stability, enduring 2000 cycles at 2 A g^−1^. Similarly, scientists successively discovered poly(benzoquinonyl sulfide) (PBQS) (Fig. 10d) with monomer with one ether bond and poly(2,3-dithiino-1,4-benzoquinone) (PDB CSO) with monomer with two ether bonds [111]. It has been demonstrated that trifluoromethanesulfonate is embedded at the ether bond. Other works also put emphasis on the green synthesis of quinon-based electrodes. Liu's group grafted polydopamine (PDA) onto carbon nanotubes to prepare the environmental-friendly cathode (Fig. 10e). The phenol–hydroxyl and quinone conversion was realized during the charge–discharge process with the insertion and removal of Zn^2+^ [116].

### Aniline-Based Linear Polymer

Polyaniline (PANI), which is the result of oxidative polymerization of aniline in acidic aqueous environments, can be oxidized to form positively charged C–N^+^ when charged, allowing it to store anions. Conversely, it incorporates with cations in the electronegative nitrogen position (C–N^−^) when reduced. It exhibits a range of oxidation states with varying doping levels and has been extensively used in various battery systems [129–133]. In aqueous metal-ion batteries, polyaniline has been used as efficient cathode due to its rich active sites and good conductivity [117–122, 134–137].

**Fig. 11:**
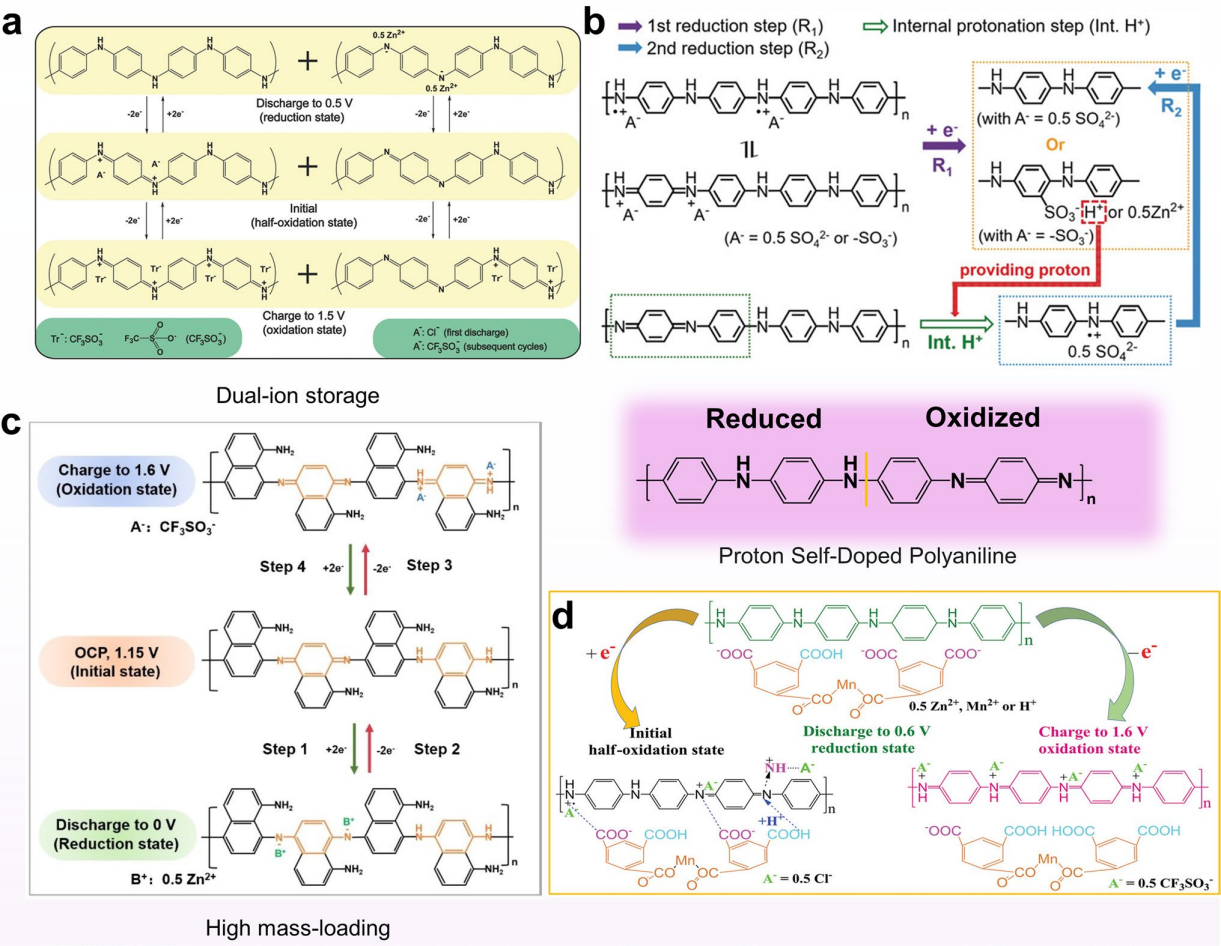
Proposed reaction mechanism of **a** PANI/CFs. Reprinted from Ref. [120] with permission from John Wiley and Sons. **b** PANI-S. Reprinted from Ref. [121] with permission from John Wiley and Sons. **c** Molecular structure of PDAN. Reprinted from Ref. [123] with permission from John Wiley and Sons. **d** PANI-M. Reprinted from Ref. [138] with permission from John Wiley and Sons

Traditional AZIBs primarily relied on the intercalation and deintercalation of Zn^2+^. However, with the advent of the dual-ion mechanism, researchers aimed to integrate it with conventional mechanisms to attain higher operating voltages and enhance battery performance. In 2018, Chen and colleagues reported the utilization of the dual-ion mechanism in AZOBs, combined with the preparation of half-oxidized PANI (Fig. 11a) [120]. During the first discharge, the = NH^+^ − gained electrons to be reduced to −NH− , and Cl^−^ was removed from PANI. The =N− was reduced to −N− , which could interact with the Zn^2+^. When charging later, the − N^−^ − in PANI was oxidized to =N− and hence the Zn^2+^ interacted with −N^−^− was removed from PANI. Calculations based on the XPS results showed that the Zn^2+^ embedding delocalization contributed about 40% of the capacity, leaving about 60% of the capacity contributed by the dual-ion mechanism. Owing to hybrid mechanism, the rate performance is more outstanding. When the current density increased from 0.05 to 5 A g^−1^, the charging and discharging plateau remained evident and the polarization voltage increased from 0.174 to 0.382 V. Even the capacity at 5 A g^−1^ remained at approximately half of that at 0.05 A g^−1^. In both cases, there were no significant dendrites on the Zn surface. Furthermore, the authors explored the potential possibilities of this battery system in the field of flexible electronics.

It has become evident that the presence of protons plays a crucial role in facilitating the redox process of polyaniline. However, the challenge lies in creating a proton-rich environment for the redox process of the PANI electrode without causing corrosion of the zinc electrode under such acidic conditions. In 2018, Sun's group introduced a sulfonyl self-doped polyaniline cathode material to address this issue [121]. They prepared the electrode named PANI-S through a simple electrochemical method of copolymerization of aniline and metanilic acid. The pKα of metanilic acid is smaller than that of zinc sulfate electrolyte so that –SO_3_H was ionized to generate H^+^, which made the local concentration of H^+^ larger and thus improved the redox reaction of polyaniline (Fig. 11b). Further, PANI-S electrode has a lower charge transfer resistance of 1.2 Ω compared to the non-doped PANI electrode, and the non-doped showed a rapid capacity decay during cycling while the PANI-S delivered a high capacity of 180 mAh g^−1^ and a long-cycle ability. Nevertheless, the inclusion of certain sulfonic acid groups proves inadequate in managing the structural alterations that transpire during charge/discharge cycles, and the deprotonation of polyaniline continues to transpire in a highly acidic setting. In an effort to address these challenges, Fang's group integrated robust MOFs to alleviate structural stress and harnessed carboxyl groups to ensure the effective supply of protons (Fig. 11d) [138]. These researches have widen strategies for optimizing polyaniline electrodes in AZIBs.

Thanks to the excellent conductivity of aniline and a well-established synthesis process, a new organic semiconductor polymer poly(1,8-diaminonaphthalene) (PDAN) was investigated to reach the commercial mass loading level (~ 10 mg cm^−2^) [123]. Similar to the charge storage mechanism of PANI, PDAN featured an extended π-conjugated structure that enhanced its conductivity to a certain extent (Fig. 11c). In the initial discharge of PDAN, =N− gained electrons, undergoing reduction to −N^−^− , enabling coordination with Zn^2+^ ions in the electrolyte. Conversely, during the initial charging, −NH− was oxidized to =NH^+^− to store the anion. As a result of these findings, PDAN could be cycled up to 1000 times under high mass loading (10 mg cm^−2^). The Zn utilization could reach 11.9% even with a loading of up to 17 mg cm^−2^, which was hardly reported in the previous studies. Therefore, despite the challenges related to proton effects, its high conductivity, feasible dual-ion storage mechanism, and capability to meet practical mass loading requirements still make it a compelling candidate for practical AZIBs.

### Nitrogen-Containing Heterocyclic Polymers

As per the definition, a nitrogen-containing heterocyclic polymer is a polymer with a ring structure that incorporates nitrogen along with carbon in the atoms forming the ring. Common examples include pyrrole, imidazole and others. Nitrogen-containing heterocyclic polymers such as polypyrrole and TEMPO derivatives have found numerous applications in AZIBs.

**Fig. 12:**
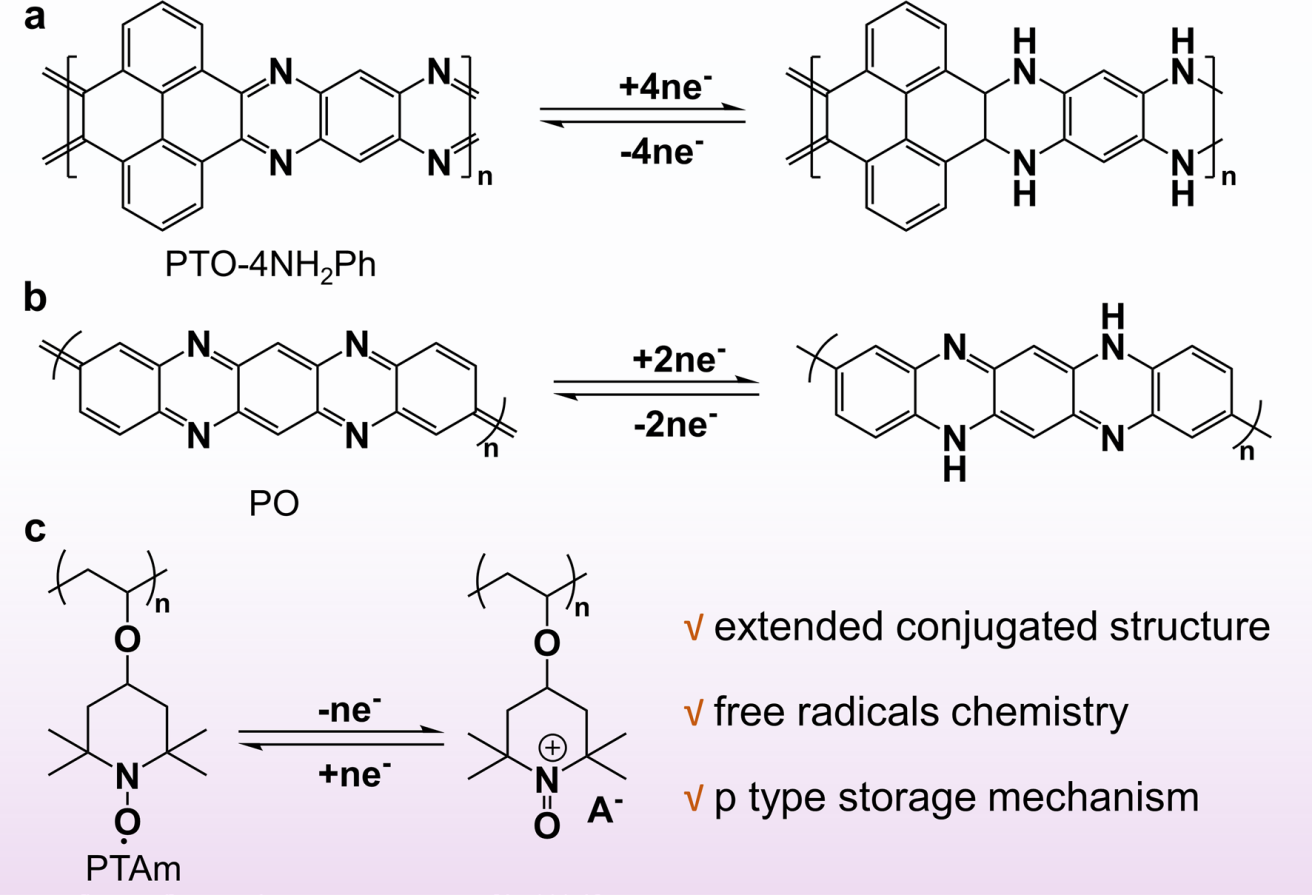
Schematic illustration of molecular engineering of nitrogen-containing heterocyclic linear polymers

Tao's group polymerized pyrene-4,5,9,10-tetraketone (PTO) and 1,2,4,5-tetraaminobenzene (4NH_2_Ph) to obtain a new polymer PTO-4NH_2_Ph (Fig. 12a) [54]. They converted the active group C=O of the PTO unit into C=N to achieve the conjugated amine structure with better conductivity, and benzene ring was used as a connecting unit to further expand the conjugated plane of the polymer. They creatively used aromatic indexes (HOMA, NICS(1)_ZZ_ and LOL-π) to evaluate the aromatization of molecule. It revealed that PTO-4NH_2_Ph exhibited high aromaticity in neutral and electronic states (PTO-4NH_2_Ph^2−^, PTO-4NH_2_Ph^4−^), contributing to better structural stability. It had a phenazine-like structure similar to PTO-4NH_2_Ph, but is a PoPD polymerized by o-phenylenediamine, and the denser C=N bonds enabled higher capacity and longer cycling life. Moreover, Niu's and co-workers designed π-conjugated poly (2,9 dihydroquinolizine[2,3-b]phenazine) (PO) [115], which was capable of a two-step redox reaction with a considerable voltage difference due to the large LUMO gap between the PO molecule and its reducing product. Notably, the C=N group guaranteed that PO molecules manifested H^+^ intercalation/detachment in the ZnSO_4_ electrolyte (Fig. 12b). Therefore, they developed a neutral all-organic proton battery, which provided implications for the design of sustainable proton batteries in neutral electrolytes. Apart from pure polymer materials, Li's group combined it with inorganic materials while increasing the carbonyl active site to synthesize a wrapped poly(m-aminophenol, 3-AP) interlayer and a well-conductive poly(p-aminophenol, 4-AP) epidermal electrodeposited nanoporous carbon, called C@poly(3-AP)/poly(4-AP) [124]. The synergistic effect resulted in C@poly(3-AP)/poly(4-AP) cathodes with ultra-high specific capacity, excellent rate performance, and long lifetime over the original C@poly (3-AP) and C@poly (4-AP) electrodes.

Another more intentional material is a linear polymer containing nitrogen and oxygen radicals. In order to broaden the voltage window, Koshika et al. reported a poly(2, 2, 6, 6-tetramethylpiperidinyloxy-4-yl vinyl ether) (PTVE) layer as cathode material for zinc-ion batteries that showed excellent rate performance and high discharge voltage platform (1.7 V) [125]. Consequently, it becomes crucial to design an aqueous electrolyte with a high stability potential window to match the high-voltage organic cathode. Luo et al. compared the effects of electrolytes containing SO_4_^2−^, CF_3_SO_3_^−^, and ClO_4_^−^ on the electrochemical performance of batteries. Three batteries with different electrolytes showed discharge voltage plates of 1.77, 1.58, and 1.53 V, respectively. DFT calculations also confirmed that the higher the binding energy of the anion and PTVE, the higher the operating voltage of the battery. However, PTVE had poor recyclability due to its slow dissolution. Immediately, the group designed a new Tempo polymer-poly(2,2,6,6-tetramethylpiperidinyloxy-4-yl acrylamide) (PTAm), which differed from PTVE in that –CONH– is substituted for –O– (Fig. 12c) [139]. In aqueous NaBF_4_ solution, PTAm presented an initial capacity of 114 mAh g^−1^ at a rate of 60C and still retained 97% after 1000 cycles.

Utilizing the intrinsic features of smart polymers, researchers have turned their interest to wearable electronic devices. Hu et al*.* proposed an electrochromic flexible aqueous zinc-ion battery using electrochromic polypyrrole (PPy) as the cathode and polyvinyl alcohol (PVA) hydrogel as the electrolyte for the first time [126]. The battery changed from black to yellow when voltage decreased from 1.2 to 0 V, which is the principle of the short-circuit warning of this battery. Notably, the battery showed a high capacity of 123 mAh g^−1^ with the bending degree is up to 180° while keeping the performance of the battery.

### Beyond Carbonyl and N-Containing Structures

**Fig. 13:**
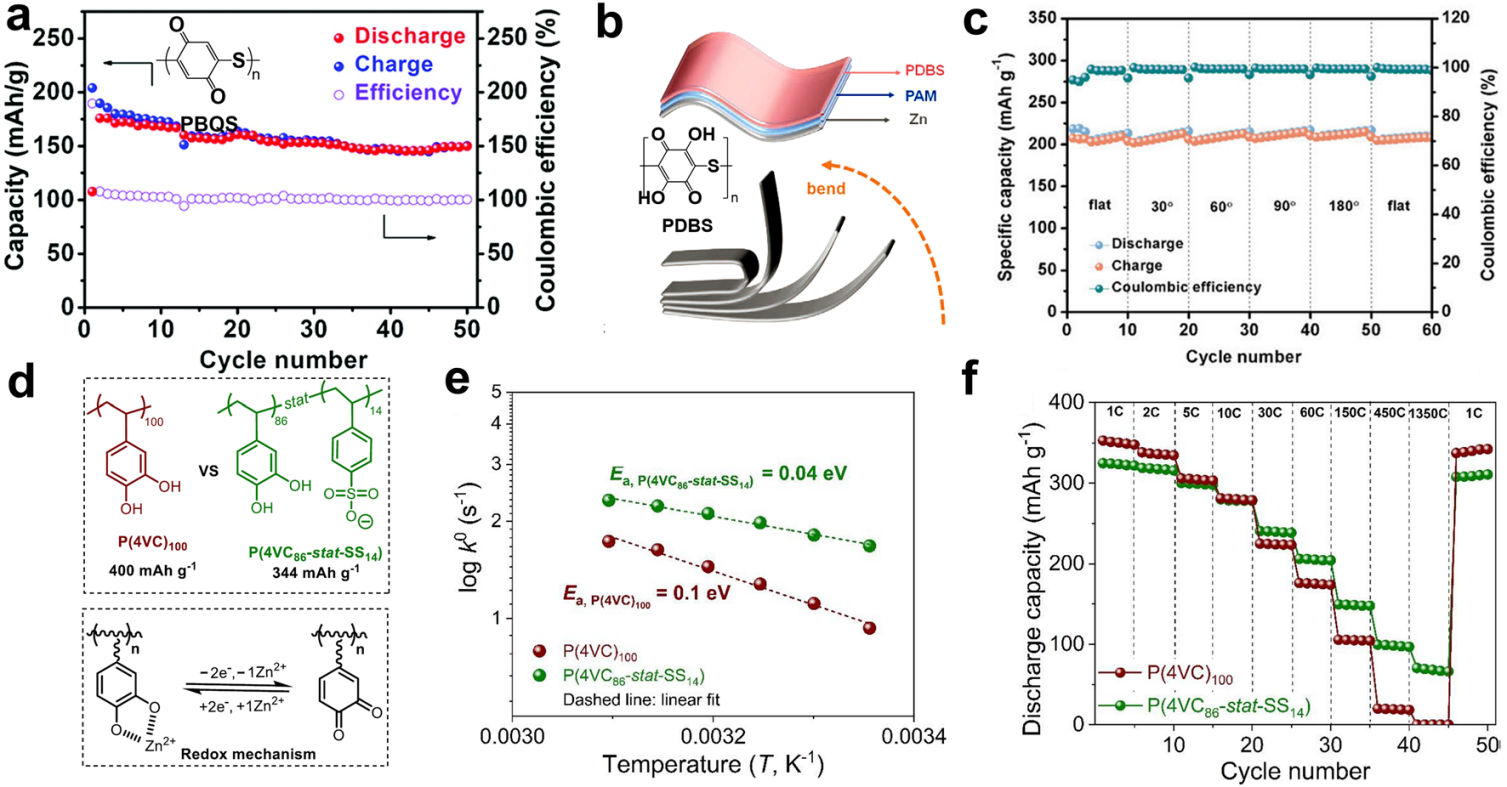
**a** Electrochemical performances of PBQS. Reprinted from Ref. [111] with permission from Royal Society of Chemistry. **b** Flexible electrodes and electrochemical performances of PDBS under bending. **c** Cycling performance under variable bending angles. Reprinted from Ref. [110] with permission from John Wiley and Sons. **d** Charge storage process and **e** a fitting of temperature dependence of rate constant with the Arrhenius equation to calculate the activation energy of P(4VC_86_-stat-SS_14_). **f** Rate capability of P(4VC_86_-stat-SS_14_). Reprinted from Ref. [109] with permission under an open access license

Thioether structures have been introduced to enhance electrical conductivity and decrease the solubility like PBQS, thus showing excellent cycling stability (Fig. 13a). The flexible property of the thioether bond itself also causes the molecular structure to twist, resulting in unique helical folded structures, such as PDBS electrode. Flexible lattice facilitates the insertion/removal of Zn^2+^; thus, PDBS shows the potential for belt-shaped flexible batteries (Fig. 13b, c). Attempts have also been made to expand planar conjugated structures to facilitate electron transport. For example, the π electrons can be well delocalized in the skeleton of reduced PTO-4NH_2_Ph, indicating great stability after being reduced. Conductive polymers like polyaniline and polypyrrole exhibit intrinsic high conductivity. When they come to polymers containing side chains, the electrochemical activity/inertia of side-chain groups needs to be considered. On the one hand, active ones such as nitroxide radicals (PTVE) could promote outstanding discharge voltage. On the other hand, electrochemical inerts such as sulfonic acid groups (P(4VC_86_-stat-SS_14_)) are intended to promote the transfer of Zn^2+^ and enhance the wettability of the electrode by improving the hydrophilic property of the polymers, which is of great importance to achieve fast kinetics and superior rate capability (Fig. 13d–f). Overall, apart from active centers, the electron and ionic conductivity of the polymer is a key issue to promote efficient redox reactions in aqueous electrolytes, which triggers the urgent development of side-chain engineering since it simply outperforms other strategies in tuning the physicochemical properties of polymers.

## Porous Polymeric Framework-Based Aqueous Cathode

CMPs and COFs have garnered significant attention due to their designable ordered structures, inherent high porosity, and exceptional physicochemical properties. The planar and three-dimensional ordered stacking structures bring about substantial surface area and numerous active sites, making them as outstanding candidates for high-performance electrodes. In contrast to COFs, CMPs lack a typical crystal structure, making the synthesis conditions comparatively less stringent. The initial work in this area was reported by Jiang's group; they introduced a series of polypyrene compounds with varying connection structures and electronic properties as organic cathode materials for aqueous zinc dual-ion batteries (AZDIBs) [140]. It is demonstrated that the electronic structure of polypyrene can be manipulated by altering the connection site on the pyrene unit within the polypyrene structure, allowing for the adjustment of its redox activity. Polypyrene CLPy, connected at the 1,3,6,8-loci, exhibited a highly delocalized HOMO distribution, a high HOMO energy level, a narrow band gap, and a large surface area. These properties allowed for a high Cl^−^ storage capacity of 180 mAh g^−1^, which was significantly superior to the other two linear polypyrene materials, LPy-1 (24 mAh g^−1^) and LPy-2 (44 mAh g^−1^). Furthermore, CLPy exhibited exceptional cycling stability, maintaining a capacity retention rate of 97.4% after 800 cycles at 50 mA g^−1^, and an impressive 96.4% even after 38,000 cycles at 3 A g^−1^. Notably, CLPy also displayed low self-discharge rates, retaining approximately 90% of its capacity after 28 days of storage. Another noteworthy development is the production of polytriphenylamine CMP (Fig. 15a), which demonstrated the capability to store energy through Cl^−^ accommodation in a pseudocapacitance-dominated fashion [141]. It exhibited a COF-like porous structure with a specific 3D conjugate network that ensured high utilization of N active sites (up to 83.2% at 0.5 A g^−1^) and unique cycling stability during repeated charge–discharge (87.6% capacity retention after 1000 cycles). The latest research is Wang group's research on the mechanism of CMP configuration on energy storage. By comparing P3Q triquinoxyl(3Q)-based homopolymer (P3Q) and a triazine-linked 3Q polymer (P3Q-t) [142], it was revealed that the storage mechanism is intricately linked to the forces acting both between and within molecules. P3Q-t exhibited high conjugated flatness and electronegative fusion loop pathways, leading to faster reaction kinetics and lower Zn^2+^ transfer resistance (Fig. 15b). As a result, P3Q displayed interactions with both Zn^2+^ and H^+^, while P3Q-t was found to selectively interact only with Zn^2+^.

**Fig. 14:**
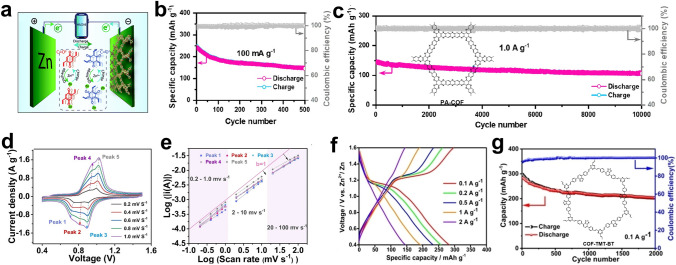
**a** Diagrammatic representation of the aqueous Zn/HqTp unit cell. Reprinted from Ref. [143] with permission under a Creative Commons Attribution-NonCommercial 3.0 Unported Licence. **b** Galvanostatic cycling performance at a current density of 0.1 A g^–1^. **c** Long-term cycling stability at 1 A g^−1^. Reprinted from Ref. [144] with permission from American Chemical Society. **d** CV curves of BT-PTO COF at multiple scan rates. **e** Relationship between log i and log v during the sweep rate 0.2–100 mV s^−1^. Reprinted from Ref. [56] with permission from John Wiley and Sons. **f** GCD profiles for COF-TMT-BT electrodes. **g** Long-term cycling performance at 0.1 A g^−1^. Reprinted from Ref. [145] with permission from John Wiley and Sons

**Fig. 15:**
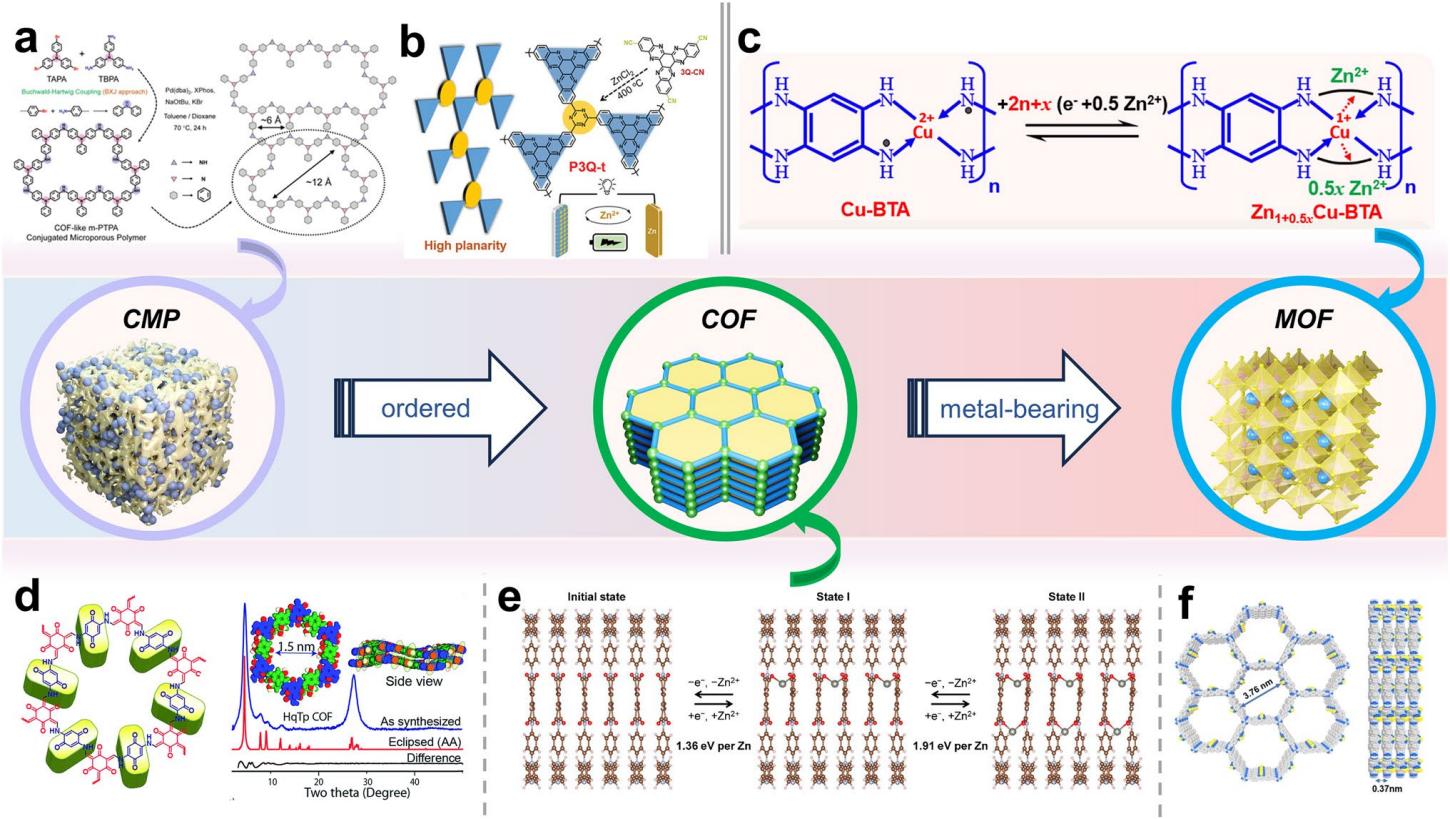
**a** Schematic illustration of the synthesis route of m-PTPA and its structure. Reprinted from Ref. [141] with permission from John Wiley and Sons. **b** Schematic illustration of synthesis, molecular planarity, and ZIB performance of P3Q-t. Reprinted from Ref. [142] with permission from John Wiley and Sons. **c** Proposed reaction mechanism for Cu-BTA-H. Reprinted from Ref. [148] with permission from American Chemical Society. **d** Schematic representation of HqTp and Powder X-ray diffraction pattern. Reprinted from Ref. [143] with permission under a Creative Commons Attribution-NonCommercial 3.0 Unported Licence. **e** Zn^2+^-storage mechanism of PI-COF simulated by DFT calculations. Reprinted from Ref. [146] with permission from American Chemical Society. **f** Top and side view of the AA eclipsed model of COF-TMT-BT. Reprinted from Ref. [145] with permission from John Wiley and Sons

COFs are constructed from pre-designed symmetrical organic building blocks and self-assemble through π–π stacking. Compared to CMPs, COFs feature long-range ordered open channels and a well-defined crystal structure. In 2019, Banerjee and colleagues introduced a novel method by mechanically mixing 2,5-diaminohydroquinone dihydrochloride (Hq) and 1,3,5-triformylresorcinol (Tp) to synthesize a COF material (HqTp), employing p-toluenesulfonic acid as a catalyst [143]. The powder X-ray diffraction (PXRD) profile exhibited sharp several peaks and the model simulated a pore size of about 1.5 nm (Fig. 15d). It revealed that the efficient interlayer interaction of these divalent Zn^2+^ ions with C=O and N–H from the adjacent layers (Fig. 14a) provided an excellent discharge capacity (276.0 mAh g^−1^ at 125.0 mA g^−1^). They also used a novel phenanthroline covalent organic framework (PA-COF) as a cathode material for AZIBs [144]. Through theoretical simulation analysis, it found that the o-phenanthroline unit is the active site that bound to zinc ions. The capacitance contribution of Zn^2+^ and H^+^ was determined by inductively coupled isosomal emission spectroscopy (ICP-OES) and solid-state nuclear magnetic resonance (NMR). Electrochemical tests presented that PA-COF had a high capacity of 247 mAh g^−1^ at a current density of 0.1 A g^−1^ and can stabilize 10,000 cycles at 1.0 A g^−1^, with an average capacity attenuation of only 0.38% per cycle (Fig. 14b, c). Under the guidance of Banerjee, Tao and colleagues modified the number and degree of unsaturation of active sites, resulting in the synthesis of a covalent organic framework (BT-PTO COF) featuring an ordered channel structure. This COF was created from benzene tricarboxylic (BT) and PTO active monomers [56]. The inherent ordered channel structure of BT-PTO COF facilitates ion transfer and insertion, resulting in superior rate and cycling performance compared to existing organic-based cathode materials in zinc-ion batteries. Kinetic analysis corroborated a redox pseudocapacitance mechanism, beginning with the insertion of Zn^2+^ followed by the co-insertion of two H^+^ (Fig. 14d, e). At high current densities, the predominant insertion pathway shifted from H^+^ and Zn^2+^ co-insertion to a greater emphasis on H^+^ insertion. This research provides a fundamental basis for the subsequent design and investigation of quinone-based covalent organic framework compounds. Additionally, Liu and colleagues synthesized Tp-PTO-COF by condensing 1,3,5-triformylresorcinol (Tp) and 2,7-diaminopyrene-4,5,9,10-tetraketone (DAPTO) [103]. Tp-PTO-COF featured a more abundant carbonyl nucleophile center, an ordered porous structure, and inherent chemical stability, all of which are favorable for Zn^2+^ ion storage and diffusion. Experimental analysis revealed that the ion intercalation mechanism aligned with theoretical simulations. Consequently, AZIBs assembled with the Tp-PTO-COF electrode demonstrated high capacity output, a flat charge–discharge platform, and outstanding cycle stability.

The amide structure also plays a role in COF electrode materials. Feng's group reported a 2D polyarylimide covalent organic framework (PI-COF) anode material with high kinetic Zn^2+^ storage capacity [146]. The broad pore structure of PI-COF offered improved ion diffusion channels and exposed a greater number of redox-active carbonyl groups. As a result, the PI-COF anode displayed a remarkable specific capacity (332C g^−1^ or 92 mAh g^−1^ at 0.7 A g^−1^), exceptional rate performance (79.8% at 7 A g^−1^), and an extended cycle life (85% retention rate after 4000 cycles). In situ Raman spectroscopy investigations, along with first-principles calculations, provided insights into a two-step Zn^2+^ storage mechanism (Fig. 15e). In this mechanism, the carbonyl group of the imide underwent reversible formation of negatively charged enolates. Similar to polymers, the greater the number of active sites in the monomer and the smaller the relative molecular weight, the higher the capacity for storing Zn^2+^ ions. Alshareef introduced a strategy to enhance the charge storage performance and discharge potential of the COF cathode. The HAQ-COF was achieved by grafting quinone onto a 1,4,5,8,9,12-hexaazatriphenylene-based COF (HA-COF) [147]. They conducted a thorough analysis of the charging and discharging mechanisms of HAQ-COF materials, including the competitive coordination process of Zn^2+^ and H^+^ within these materials. The experimental findings demonstrated that, at a current density of 0.1 A g^−1^, the material exhibited a specific capacity of 344 mAh g^−1^, and when the current density was increased to 5 A g^−1^, the cycle stability significantly improved, with a capacity retention rate of 85% after 10,000 cycles. Theoretical calculations revealed that reducing the LUMO energy level of HAQ-COF materials can increase the discharge potential and thus increase the energy density. In addition, Zn^2+^/H^+^ preferentially coordinates with C=O and C=N. The experimental analysis of charge storage mechanism further verified the proposed favorable redox activity center carrying Zn^2+^/H^+^ on COF electrode. This study exemplified an effective approach for designing COF structures at the molecular level, offering a viable strategy for enhancing their electrochemical performance. However, an excessive number of carbonyl groups can elevate the solubility of COF materials. Consequently, Artur's group introduced a novel type of robustly structured olefin-linked COF-TMT-BT as a cathode. This COF was synthesized through the condensation of aldehydes between 2,4,6-trimethyl-1,3,5-triazine (TMT) and 4,4′-(benzothiadiazol-4,7-diyl)dibenzaldehyde (BT), featuring benzothiadiazole units as new electrochemically active groups [145]. The COF-TMT-BT exhibited large pore size and outstanding Zn^2+^ storage capacity (Fig. 15f), delivering a high capacity of 283.5 mAh g^−1^ at a current density of 0.1 A g^−1^ (Fig. 14f, g). Calculations and experimental analysis revealed that the charge storage mechanism of the COF-TMT-BT electrode relies on supramolecular engineering and the reversible coordination of Zn^2+^ with benzothiadiazole units.

Compared to COFs, the intrinsic multivalent valence states of metals in metal–organic frameworks (MOFs) contribute to the high activity and reversibility of their redox pairs. Liang et al. developed a one-dimensional π-d-conjugated MOF through the coordination of tetraaniline and copper ions. Through ex situ XPS spectra and the calculation of sequential binding energy, they proposed a reaction mechanism depicted in Fig. 15c: involving the reversible transformation of Cu^2+^/Cu^+^ and –C═N/C–N to store zinc ions [148]. The high redox potential of Mn^3+^/Mn^2+^ (1.51 V vs. SHE) has garnered significant interest. Liu et al. employed polyacrylic acid with molecular weights ranging from 3 to 7 million as a ligand to stabilize this Mn ion pair in a mild environment [149]. The CV curves exhibit a peak at 1.83/1.67 V, which is attributed to Mn^3+^/Mn^2+^. In the GCD curves, the interval of 1.4–1.9 V shows higher capacity retention in comparison with the range of 0.8–1.9 V, indicating good reversibility of Mn^3+^/Mn^2+^. The carriers were confirmed to be protons through FTIR and in situ pH monitoring of the cathode surface.

**Fig. 16:**
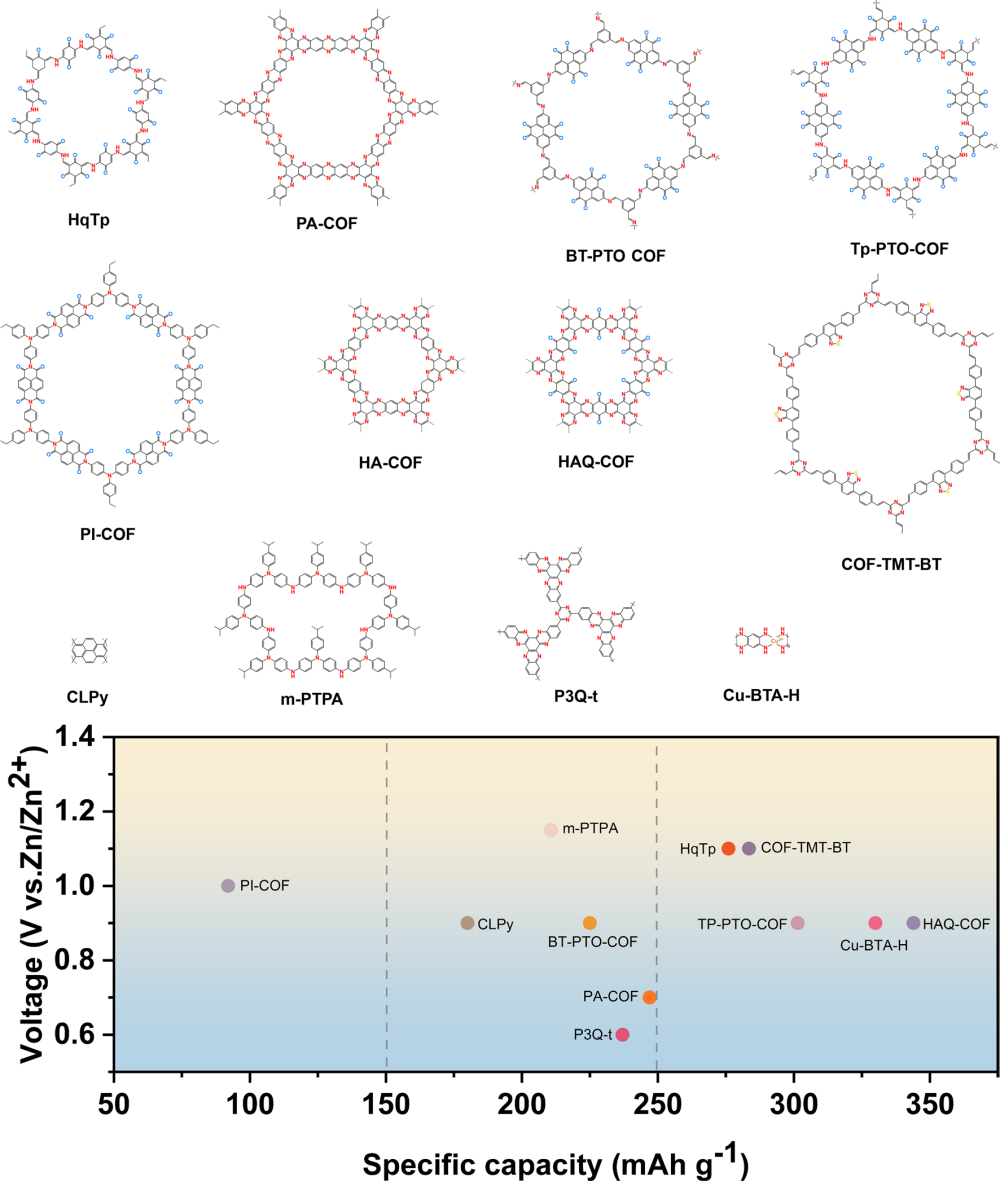
Molecular structures of representative porous organic electrode for AZIBs and comparison of their capacity and voltage

Figure 16 and Table 3 summarize the representative examples with their corresponding electrochemical performances. Although COFs with ordered porous structure unique conducting features promote their application in AZIBs, great challenge in facile synthesis has hindered their practical application at present. CMP materials are comparatively easier to synthesize than COFs, but their amorphous nature may lead to overlapping pore sizes and the potential obstruction of active sites. MOFs bear the advantage of highly ordered structure and the introduction of metal ions that can result in unexpected redox potentials. Specifically, two-dimensional MOFs exhibit excellent conductivity owing to their graphene-like structure. However, the electrochemical stability of MOFs often falls short due to the weak coordination bonds. Besides, their synthesis and attainment of a single-crystal structure also pose significant challenges. Consequently, devising a green, environmentally friendly, cost-effective, and expeditious synthesis method for porous polymer electrode materials is an urgent problem in need of resolution.

**Table 3:** Summary of electrochemical properties of polymer with framework structure

#	Charge carriers	Cathode	Anode	Electrolyte	Output voltage (V)	Cycling stability	Specific capacity(mAh g^−1^)	Refs
1	Zn^2+^	HqTp	Zn	3 M ZnSO_4_	~ 1.1(0.2–1.8)	95%/1000/3.75 A g^−1^	276.0 at 125.0 mA g^−1^	[143]
2	Zn^2+^ and H^+^	PA-COF	Zn	1 M ZnSO_4_	~ 0.7	0.38% decay per cycle/10000/1 A g^−1^	247 at 0.1 A g^−1^	[144]
3	Zn^2+^	BT-PTO COF	Zn	3 M Zn(CF_3_SO_3_)_2_	0.8 ~ 1.0	98.0%/10000/5 A g^−1^	225 at 0.1 A g^−1^	[56]
4	Zn^2+^	Tp-PTO-COF	Zn	2 M ZnSO_4_	0.8 ~ 1.0	95%/1000/2 A g^−1^	301.4 and 192.8 at current densities of 0.2 and 5 A g^−1^, respectively	[103]
5	Zn^2+^	MnO_2_	PI-COF	2 M ZnSO_4_	0.06 − 0.96 V vs Zn^2+^/Zn	85%/4000/4 mv s^−1^	92 at 0.7 A g^−1^	[146]
6	Zn^2+^ and H^+^	HAQ-COF	Zn	2 M ZnSO_4_	0.8 ~ 1.0	85%/10000/5 A g^−1^	344 at 0.1 A g^−1^	[147]
7	Zn^2+^ and H^+^	TTPQ	Zn	2 M ZnSO_4_	1.07	94%/250/0.5 A g^−1^	404 at 0.3 A g^−1^	[150]
8	Zn^2+^	COF-TMT-BT	Zn	2 M Zn(CF_3_SO_3_)_2_	0.5 ~ 1.6	65.9%/2000/0.1 A g^−1^	283.5 at 0.1 A g^−1^	[145]
9	Zn^2+^ and Cl^−^	CLPy	Zn	30 M ZnCl_2_	~ 0.9	96.4%/38000/3 A g^−1^	180 at 50 mA g^−1^	[140]
10	Zn^2+^ and Cl^−^	m-PTPA	Zn	2 M ZnCl_2_	1.15	87.6%/1000/6 A g^−1^	210.7 at 0.5 A g^−1^	[141]
11	Zn^2+^	P3Q-t	Zn	2 M ZnSO_4_	~ 0.6	81%/1500/3 A g^−1^	237 at 0.3 A g^–1^	[142]
12	Zn^2+^	Cu_3_(HHTP)_2_	Zn	3 M Zn(CF_3_SO_3_)_2_	~ 0.9	75%/500/4 A g^−1^	228 at 50 mA g^–1^	[151]
13	Zn^2+^	Cu-BTA-H	Zn	2.5 M ZnSO_4_	~ 0.9	68.5%/500/2 A g^−1^	330 at 200 mA g^–1^	[148]
14	H^+^	PAL-Mn	Zn	1 M ZnSO_4_ + 0.5 M MnSO_4_	1.67	100%/4000/1 A g^−1^	90 at 1 A g^–1^	[149]

## Structural Effect of Organic Electrode Materials

**Fig. 17:**
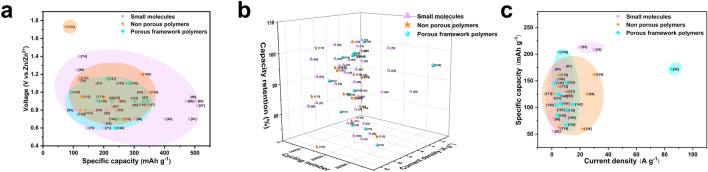
Diagrams of the electrochemical performances of representative organic materials for AZIBs in terms of **a** specific capacity, **b** long-term cycling performance, and **c** rate capability

We have compiled and illustrated comparative diagrams of the aforementioned organic materials for AZIBs across three different aspects: specific capacity, rate capability, and long-term cycling performance (Fig. 17). It can be observed that the specific capacity of AZIB cathode materials mostly falls within the range of 100–350 mAh g^−1^. Compared to electrode materials with similar molecular structures in LIBs, they exhibit relatively lower performance, typically achieving around 70% of the theoretical capacity. Firstly, Zn^2+^, being divalent cations, experience significant electrostatic repulsion. Secondly, the larger size of Zn^2+^ impedes rapid ion exchange and transfer compared to lithium ions. Additionally, during the intercalation/deintercalation process, Zn^2+^ tend to bind with hydration molecules, leading to volumetric changes that can disrupt the electrode structure and diminish its capacity. Thirdly, Zn^2+^ may undergo unpredictable parasitic reactions, forming unstable or irreversible intermediates during their intercalation/deintercalation, thereby compromising electrode reversibility, capacity, and lifespan.

Small molecules generally exhibit relatively high tested capacities and broader voltage range. Some well-conjugated small molecules with both pyrazine and quinone moieties can even achieve specific capacities of around 500 mAh g^−1^ at low currents. This advantage stems from their excellent planar conjugation, good conductivity conferred by the D-A structure, poor water solubility, and the presence of dense active sites. Additionally, the introduction of quasi-pyrazine structures to some extent enhances the insertion and extraction of H^+^ between molecular structures, while the synergistic effect of H^+^ and Zn^2+^ accelerates the reaction kinetics. However, due to the inherent solubility of small molecules, their electrochemical stability is compromised. Consequently, when the shuttling of Zn^2+^ and H^+^ ions occurs rapidly, relatively low capacity retention rates under high currents are resulted. Modern electronic devices require both fast charging/discharging capabilities and long-term durability; hence, improving rate capability and long cycling performance is imperative. Non-porous polymer materials principally address the dissolution issue by incorporating active small molecules into side chains or polymerizing them as polymer units, thereby significantly enhancing long-term cycling performance. Moreover, polymerization expands the conjugated system of small molecules, reducing the energy gap between the homo and lumo orbitals and enhancing conductivity. Compared to the pre-polymerization state, both the maximum tolerable current and the capacity retention rate under the same current can be significantly improved for the electrode material. However, the uncontrollability of polymerization leads to unpredictability in the polymerization degree and the twisting arrangement of carbon chains, resulting in the burial of some active sites. Besides, the large content of covalent bond formed by polymerization reaction increases the dead weight of the electrode material, thereby exhibiting lower theoretical and tested capacities. Some poorly conjugated porous polymers can only achieve around 50% of their theoretical capacity.

Therefore, designing open-framework structures to accelerate electron and ion transport is crucial. CMPs with high surface area exhibit rapid ion diffusion kinetics owing to their unique porous nature. Moreover, their active sites are maximally exposed, facilitating rapid ion binding with electrolytes. Highly crystalline COF materials, characterized by orderly open channels, abundant pores, and stable structures could enable excellent rate capability. They are expected to achieve considerable specific capacities, high-rate and long-term cycling performances. However, the close stacking layers in COFs and CMPs electrode materials limit the utilization of active sites, preventing them from reaching the theoretical capacities seen in small molecules. In this regard, exfoliating 2D COFs/CMPs into monolayers or constructing 3D porous frameworks become efficient approaches to induce sufficient exposure of active sites, however bringing about additional challenges for post-treatment.

The output voltage, a critical determinant of battery energy density, is primarily dictated by the characteristics of electrode materials and electrolytes. The redox reactions involving pyrazine structural units exhibit multiple peak formations between 0.6 and 1 V, whereas quinone units predominantly peak near 1 V. As a result, conventional n-type materials, in common electrolyte systems, often exhibit output voltages around 0.6 to 1.1 V. This is attributed to the intentional introduction of strong electron-withdrawing groups such as –F, –Cl, –Br, –C=O, and –CN during molecular design to lower the LUMO energy level and consequently elevate the output voltage. Conversely, densely packed small molecules and certain polymer materials could increase their respective electron-withdrawing capabilities by increasing the intermolecular distance between functional groups, thereby enhancing the output voltage to some extent. Another approach involves designing polymer frameworks containing p-type structures. However, the occurrence of water electrolysis reactions at lower potentials restricts the realization of high-voltage reactions. Hence, research on high-voltage electrolytes for AZIBs remains crucial. Strategies such as high-concentration electrolytes or organic additives like acetonitrile and cyclodextrin molecules to reduce free water in the electrolyte could be pursued in this regard.

## Summary and Perspectives

In summary, AZIBs based on organic electrodes offer a promising solution to the anticipated surge in demand for green EES with promising application in grid energy storage and so on. The design principle of these organic electrodes become extremely crucial in respects of high efficiency, low cost, scalability, and degradability from the commercialization point of view. As depicted in this review, the structural–property relationship is inspiring for the rational design of organic AZIBs. Integrated structural design with efficient active centers and appropriate molecular size/geometry would be a practical way to improve the electrochemical performances.

Small molecules with high utilization of active sites but severe dissolution and poor thermostability bring about a trade-off between high initial capacity and stable cycling. Increasing the conjugation and planarity of organic small molecules and hybridization with inorganic components show their effectiveness in tackling the above-mentioned problem. Notably, the synthesis of small molecules is relatively simple and suitable for mass production and hence the practical application. For polymeric electrode materials, the hindrance of steric bulk often leads to low utilization of active materials. However, due to its possible extended conjugation, multiple covalent bonds, large size and molecular weight, its rate capability, cycling stability and thermal stability can principally outperform small molecules. Besides, linear polymers containing a main chain with high conductivity and a side chain perform ether as highly active centers or ionic conductors can promote the redox kinetics by facilitated ion storage and accelerated diffusion of the electrolyte. Polymers with porous framework structure draw lessons from the above two materials. With the capability of using devisers’ monomers, linkages, and various synthetic routes, the development of CMPs, COFs, MOFs with specific active sites and variable functionality has become available. In principle, the extended 2D/3D conjugation and strong connection of monomers via covalent bond enable outstanding rate capability, thermostability and chemical stability, promoting excellent battery performances. Their long-range ordered porous structure allows efficient mass transport, which not only provides moving channels for large ions, but also facilitates sufficient wetting of electrolytes. Typically, the high porosity and specific surface area of mesoporous materials can also play a key role even if aggregated powders are formed. In this concern, high utilization of active centers is expectable by delicate tuning of the pore size and orientation. Therefore, rational design of highly efficient organic electrodes should pursue an optimization of the chemical and physicochemical properties, which calls for an integration of highly active cites, sufficient exposure of redox centers, efficient diffusion of electrolyte, and fast transfer of ions and electrons. The design of each of these features could be inspired from small molecules and polymers with different shape/geometry. It is worth noting that in-depth mechanism study is crucial to direct the rational design of organic electrodes. In this regard, multi-dimensional characterization methods can be adopted, such as some ex situ and in situ/operando analysis, which can fully reveal the real electrochemical process.

Despite the aforementioned advantages, some intrinsic drawbacks such as poor electronic conductivity and thermal stability have become long-standing obstacles for their practical use. The inherent low conductivity hinders fast transport of electrons and prohibits the full release of capacity. Besides, the harsh conditions required for the formation of polymeric frameworks still place a trade-off between high performance and scalable and low-cost fabrication. Uptake of active polymers from biomass provides a potential way to tackle the problem lies in the conventional polymerization, which brings about its sustainable nature for green energy storage. In practice, other factors beyond electrode materials also need to be considered. The narrow voltage window of the aqueous electrolyte and the dendrites of the zinc anodes in AZIBs are seriously related to the safety, stability, and energy density. At present, the mainstream charge storage mainly involves Zn^2+^ storage, Zn^2+^ and H^+^ co-intercalation and deintercalation, H^+^ storage, anion storage, Zn^2+^, and anion storage. Among them, Zn^2+^ storage is the majority, inevitably causing damage to the structure of the material. Besides, there is still no direct solution to the formation of zinc dendrites. Although the storage of protons can slow down this harmful process, the parasitic impact on the Zn anodes and other possible damage to active materials is not negligible. Due to the limitations of the electrolyte system and voltage window of AZIBs, most of the electrode materials do not involve the anion insertion behavior, which usually requires relatively higher potential than direct Zn^2+^ binding. The common strategy is to use organic additives, water-in-salt electrolyte, or mixed electrolyte to extend the voltage window. In this respect, exploiting of hydrophilic bipolar organic molecules bearing anion storage under 2 V becomes an effective way for high-performance AZIBs. In addition, although many different active sites such as ethynyl, nitro, nitroxide radicals, and sulfur atoms, have been reported, most materials are inseparable from carbonyl and phenazine groups, suggesting that it is urgent task to develop novel active materials that can efficiently adopt multi-electron reaction.

In conclusion, rapid development of green EES based on AZIBs is highly expected with the fully utilization of the collective merits of organic electrodes. It is hoped that this review can offer insight into the development of highly efficient organic electrodes for AZIBs.
